# Animal Models in the Pathophysiology of Cystic Fibrosis

**DOI:** 10.3389/fphar.2018.01475

**Published:** 2019-01-04

**Authors:** Anna Semaniakou, Roger P. Croll, Valerie Chappe

**Affiliations:** Department of Physiology and Biophysics, Faculty of Medicine, Dalhousie University, Halifax, NS, Canada

**Keywords:** cystic fibrosis, CF mice, animals in CF, CF ferrets, CF pigs, CF rats

## Abstract

Our understanding of the multiorgan pathology of cystic fibrosis (CF) has improved impressively during the last decades, but we still lack a full comprehension of the disease progression. Animal models have greatly contributed to the elucidation of specific mechanisms involved in CF pathophysiology and the development of new therapies. Soon after the cloning of the CF transmembrane conductance regulator (*CFTR*) gene in 1989, the first mouse model was generated and this model has dominated *in vivo* CF research ever since. Nonetheless, the failure of murine models to mirror human disease severity in the pancreas and lung has led to the generation of larger animal models such as pigs and ferrets. The following review presents and discusses data from the current animal models used in CF research.

## Introduction

Cystic fibrosis, is the most common lethal autosomal recessive disorder in the Caucasian population, affecting one in 2,500 to 3,000 newborns and 70,000 people worldwide (C. G. App, Epidemiology). In 1938, CF was differentiated from celiac disease and named “cystic fibrosis of the pancreas” by Andersen (1938; Davis, 2006). Paul di Sant’ Agnese, discovered the sweat electrolyte defect, and the standardization of the sweat test in 1959 made the diagnosis of CF accurate (Gibson and Cooke, 1959; Wine, 2010). Thirty years later, Riordan et al. (1989) discovered the gene responsible for CF, (*CFTR*), located on human chromosome 7. In 1989, they established the basic defect to be in a cAMP-regulated chloride channel (Welsh and Smith, 1993). Since then, an explosion of basic and clinical research has led to therapeutic progress, and the median age of survival today ranges from 40 to 53 years old, with the highest in Canada, which is a remarkable improvement from only 6 months in 1938 (Riordan et al., 1989; Rommens et al., 1989).

In addition, CF is caused by the dysfunction or absence of the CFTR channel from the exocrine tissues due to mutations in the gene (Riordan et al., 1989). While more than 2,000 mutations have been reported to date, the most common disease-causing mutation, occurring in 70% of patients, is F508del, a deletion of three nucleotides that results in the loss of phenylalanine (F) at the 508th position on the protein.

Respiratory failure is the major cause of death but as a multiorgan disease, CF also causes PI, intestinal obstruction and malabsorption, biliary cirrhosis and congenital bilateral absence of the vas deferens (Durie and Forstner, 1989; Wong, 1998; van der Doef et al., 2011; Wilschanski and Novak, 2013; Lavelle et al., 2016).

Animal models are essential tools for understanding and comprehensively investigating CF pathophysiology. Each model is unique and variably resembles human CF manifestations with variable ortholog CFTR protein identity (Table 1). This review describes the availability of various animal models for CF and how useful they are for studying disease manifestation and progression in the lungs, intestines and other affected organs.

**Table 1:** Amino acid identity in CFTR orthologs.

Species	CFTR aa Identity vs. humans	Reference
Mouse	78%	Tata et al., 1991
Pig	93%	Ostedgaard et al., 2007
Ferret	92%	Fisher et al., 2012
Rat	75.5%	Trezise et al., 1992
Zebrafish	55%	Liu et al., 2017
Sheep	91%	Harris, 1997


*Percentage of amino acid identity of CFTR orthologs from animal models described in this review, and compared to human CFTR.*

## Murine Models in Cystic Fibrosis Research

The first genetically modified mouse models of CF disease were created 3 years after the identification of the *CFTR* gene by homologous recombination in embryonic stem cells through targeting a mutation to a specific site in the murine genome (Clarke et al., 1994; Gosselin et al., 1998). Cloning of the mouse homolog of the human *CFTR* gene, which is located on mouse chromosome 6, revealed a 78% AA sequence homology to the human CFTR protein (Tata et al., 1991; Ellsworth et al., 2000). To our knowledge, 14 more mouse models have been described in the literature to date with different characteristics, grouping them in two categoriess (Table 2) (O’Neal et al., 1993; Ratcliff et al., 1993; Dorin et al., 1994; Colledge et al., 1995; Hasty et al., 1995; van Doorninck et al., 1995; Zeiher et al., 1995; Delaney et al., 1996; Rozmahe et al., 1996; Fisher et al., 2012). The first category consists of those models in which the *CFTR* gene was disrupted using a “replacement strategy” or an “insertional strategy.” A replacement strategy causes an interruption of the *CFTR* gene and generates complete nulls (“knockout”: KO) with no normal CFTR protein production, while with the “insertional strategy” a low amount (∼10%) of normal mouse CFTR mRNA is produced (Davidson and Dorin, 2001; Scholte et al., 2004). The models generated by an “insertional strategy” method are also referred as “residual function” models (Davidson and Dorin, 2001). Phenotypic differences are observed between the absolute nulls and the residual function models due to the low amount of normal *CFTR* expression in the latter.

**Table 2:** Murine models used in cystic fibrosis research.

CF mice models	Cftr mRNA	Survival to maturity	Reference
**Knockout models** (replacement method)			
*Cftr*^tm/Unc^	No WT mRNA detectable	<5%	Snouwaert et al., 1992
*Cftr*^tm/Cam^		<5%	Ratcliff et al., 1993
*Cftr*^tm/Hsc^		25%	Rozmahe et al., 1996
*Cftr*^tm3Bay^		40%	Hasty et al., 1995
*Cftr*^tm3Uth^		25%	Wilke et al., 2011
**Residual function models** (insertional method)			
*Cftr*^tm/Hgu^	10% of WT	90%	Wilke et al., 2011
*Cftr*^tm/Bay^	<2% of WT	40%	O’Neal et al., 1993
**F508del models** (replacement-gene targeting method)			
*Cftr*^tm2Cam^	30% of WT	<5%	Colledge et al., 1995
*Cftr*^tm/Kth^	Decreased levels of mutant mRNA in intestine	40%	Zeiher et al., 1995
**F508del models** (Hit and Run method)			
*Cftr*^tm/Eur^	Normal level of mutant mRNA	90%	Rozmahe et al., 1996
**Models with other mutations**			
*Cftr*^tm2Hgu^ (G551D- replacement-gene targeting method)	50% of WT	65%	Wilke et al., 2011
*Cftr*^tm3Hgu^ (G480C- Hit and Run method)	Normal level of mutant mRNA	90%	Wilke et al., 2011
*Cftr*^tm2Uth^ (R117H- replacement-gene targeting)	5–20% of WT	95%	Wilke et al., 2011
**Transgenes**			
Tg (FABPCFTR): correction of intestinal obstruction	–	1.5–8.5 months	Zhou et al., 1994
Tg (CCSPSsnn 1b): βENaC over-expression (model of CF-lung disease)	–	40%	Wilke et al., 2011
VIP-KO: CF-like phenotype in lung and intestine	Normal level of WT	Normal	Alcolado et al., 2014


*Levels of Cftr mRNA expression, compared to WT Cftr mRNA, are indicated when known. Survival rates at maturity refers to after weaning.*

The second category contains mouse models that replicate known clinical mutations in CF, such as the Class II F508del (*Cftr*^tm1Eur^, *Cftr*^tm1Kth^, and *Cftr*^tm2Cam^) and Class III, G551D (*Cftr*^tm1G551D^) (Delaney et al., 1996; Lavelle et al., 2016) utilizing a replacement-gene targeting strategy or a double homologous recombination method, also known as a “hit and run” procedure (Hasty et al., 1991; Davidson and Dorin, 2001). In this category, survival rates, disease severity and pathology vary from model to model. The variations are attributed to different types of mutations, levels of mRNA expression, environmental factors and genetic backgrounds that can be responsible for various levels of activity of modifier genes in the mouse strains used (Tarran et al., 2001; Scholte et al., 2004). Moreover, because CF mutant mice are less fertile than normal mice, breeding CF mutant mice from heterozygous animals is strongly recommended to avoid phenotypic abnormalities and experimental variability (Zeiher et al., 1995). Lastly, gender and age are factors that should be considered especially for studying the progression of lung disease.

In a recent study, McHugh et al. (2018a) generated a G542X CF mouse model with CRISPR/Cas9 gene editing method. 40.9% of founder mice expressed the G542X mutation and this new clone was generated in approximately 3 months, whereas traditional methods using embryonic stem cells, would necessitate 1–2 years to obtain the desired mouse colony. These mice have decreased CFTR expression and a lack of CFTR function in the airways and intestine; they have low survival rates due to intestinal obstruction. Pharmacological read-through of the G542X mutation in this mouse model enables functional CFTR protein production, making this model very interesting for further investigation of non-sense mutations (Than et al., 2016; McHugh et al., 2018b).

### Do Cystic Fibrosis Mice Develop Spontaneous Lung Disease?

Progressive lung disease with respiratory failure is a major clinical concern that causes 95% of the morbidity and mortality in patients with CF. Lung disease develops over recurrent exacerbations of pulmonary infections caused by a large spectrum of bacterial species including *Staphylococcus aureus*, *Haemophilus influenza*, *Pseudomonas aeruginosa* and the *Bcc* (Tarran et al., 2001; Festini et al., 2006). *P. aeruginosa* is persistent in human lungs with CF and chronic pulmonary infection is inevitable, despite antibiotic therapies, with biofilm-forming cells of a mucoid phenotype in more severe advanced infections. Spontaneous colonization with the above pathogens has not been detected in mice models unless a pathogen challenge was applied.

Kent et al. (1997) designed a model from a single genetic background (congenic), C57BL/6J *Cftr*^tm1Unc^/*Cftr*^tm1Unc^, that under specific pathogen-free conditions, developed spontaneous and progressive lung disease (Guilbault et al., 2005, 2006). Loss of mucociliary transport, post-bronchiolar hyperinflation of alveoli, and parenchymal interstitial thickening with indication of fibrosis and inflammatory cell recruitment characterize the lung disease in this congenic model (Kent et al., 1997). It also showed reduced control of the *P. aeruginosa* infection. Acinar and alveolar over inflation, reflect small airway obstructions that are observed in the early stages of CF in humans (Guilbault et al., 2005). Later on, more studies boosted our knowledge with significant observations in CF pulmonary pathology using mice that were not exposed to pathogens. Thus, excessive inflammation was noticed in *Cftr*^tmr1Hgu^ mice, MCC dysfunction in *Cftr*^tm1Hgu^ and *Cftr*^tm1Unc^ mice, goblet cell hyperplasia with ASL depletion in the nasal epithelium of *Cftr*^tm1Unc^ mice, and a distal extension of SMGs in *Cftr*^tm1Hgu^ and *Cftr*^tm1G551D^ mice (Cowley et al., 1997; Zahm et al., 1997; Tarran et al., 2001; Scholte et al., 2004). The *Cftr*^tm1G551D^ model demonstrated defective pulmonary clearance and a high sensitivity to *P. aeruginosa* (McMorran et al., 2001).

To date, many research groups using congenic mice are still trying to develop methods for *P. aeruginosa* inoculation that would mimic chronic infection with this persistent pathogen. Techniques such as intratracheal inoculation of bacteria-loaded agar beads or seaweed alginate must be used to reproduce the human lung phenotype with *P. aeruginosa*.

The fact that CF mice do not develop airway’s infections led Shah et al. (2016) to investigate the reasons why mice have functioning airway’s host defenses compared to humans and pigs with CF. This interesting study demonstrated that, in addition to the presence of Ca^+^-activated anion channels, mice also express small amounts (1–10%) of the non-gastric H^+^/K^+^ adenosine triphosphatase ATP12A transcripts, which catalyzes the hydrolysis of ATP coupled with the exchange of H^+^ and K^+^ ions across the plasma membrane. Consequently, mouse airways, including those in CF models, secrete low levels of H^+^ maintaining the pH of the ASL unaltered. The authors showed that the over-expression of ATP12A in CF mice airways caused acidification of the ASL, impairment of airway host defenses as well as increased amount of bacteria. These data suggest ATP12A as a potential therapeutic target for CF disease treatment (Shah et al., 2016).

Durie et al. (2004) described a promising long-living congenic *Cftr^-/-^* model bred in the C57BL/6 background that displays the human lung pathology with signs of inflammation, presence of macrophages, tissue damage, and acinar dilation (Durie et al., 2004). Defective bacterial clearance, increased levels of pro-inflammatory factors, and inflammation was demonstrated using *Cftr*^tm1Unc^ and *Cftr*^tm1G551D^ mice (Heeckeren et al., 1997; McMorran et al., 2001; Van Heeckeren et al., 2006). Macrophages are the most commonly found immune cells in the lung that play a significant role in bacteria eradication from the airways through phagocytosis. The input of macrophages polarization and plasticity in CF disease progression is still undefined. Studies in CF mice have shown that macrophages are unable to degrade internalized pathogens such as *P. aeruginosa* and *Bcc* (Bruscia and Bonfield, 2016; Li et al., 2017). More specifically, *Cftr^-/-^* mice challenged with *P. aeruginosa* had increased IL-8, IL-6, and TNF-alpha levels and decreased IL-10 level, indicating that the absence of CFTR can cause excessive lung inflammation (Bruscia and Bonfield, 2016). Additionally, the number of both alveolar and peritoneal macrophages was high in F508del mice treated with LPS, illustrating their adaptability in the airway environment (Bruscia and Bonfield, 2016). CF mice may not be able to develop acute lung disease as patients with CF do, but they have been successfully used to demonstrate a defective autophagy mechanism which can lead to lung hyper-inflammation (Luciani et al., 2010). An increasing amount of literature on CF macrophages is becoming available (Andersson et al., 2008; Bruscia et al., 2009, 2011; Meyer et al., 2009; Abdulrahman et al., 2011; Sorio et al., 2016; Li et al., 2017).

Zhou et al. (2011) generated a transgenic “β-ENaC” mouse model to recapitulate the pathophysiology of the CF lung. This model, which over-expresses the β subunit of the ENaC with increased Na^+^ absorption, presented with ASL depletion, reduced MCC with airway mucus obstruction, goblet cell hyperplasia, chronic lung inflammation, and high mortality related to lung disease (Zhou et al., 2011). Nonetheless, even this interesting model failed to replicate the spontaneous bacterial infection that develops in patients with CF (Zhou et al., 2011).

Overall, while the above-mentioned methods and murine models further our understanding of CF lung abnormalities, they do not provide any information on how the disease starts and the development of early stages of infection and inflammation. Furthermore, the general notion that mice do not show “spontaneous” lung pathology could be misleading. Many factors play a role in this. The short life span of mice and clean or sterile environment under which the experiments are conducted are not comparable with the conditions for human lungs with CF that are continuously challenged with various pathogens along their lifetime. In addition, diverse genetic background is an important factor, while low levels of residual CFTR protein expression in some models affect the disease severity. Moreover, the size of the airways and the presence of a non-CFTR calcium-activated Cl^-^ channel (CACC) in parallel with CFTR and other dissimilarities in lung disease between humans and mice models (Clarke et al., 1994; Gosselin et al., 1998; Lavelle et al., 2016) can account for differences in resistance to bacteria.

### Submucosal Glands to Study Fluid Secretion Defects

Submucosal glands lining the cartilaginous airways are responsible for maintaining sterility in the airways by secreting antimicrobial defenses, such as lysozyme, lactoferrin, defensins, bicarbonate, and others (Salinas et al., 2005; Ianowski et al., 2007). Moreover, they secrete mucus when stimulated with Ca^+^ -elevating agonists, for example acetylcholine (ACh) or carbachol (CCh), and/or cAMP agonists, such as forskolin (FSK) or VIP, and contain serous cells that express high levels of CFTR (Borthwick et al., 1999). In CF airway glands, the synergy between cAMP and calcium signaling is lost, and glands do not respond to VIP or FSK. In humans, SMGs extend into the bronchi, while, in mice they are confined to the trachea. Congenic mice models, especially those with a C57BL/6 genetic background, have been used to study SMG dysfunction since these mice show increased airway abnormalities and are prone to infection with *P. aeruginosa.* Grubb and Boucher (1999) mentioned that mice have very few SMGs (Grubb and Boucher, 1999). However, in a study of tracheal SMG secretion in *Cftr^-/-^* mice, Ianowski et al.(2007) observed that the upper trachea contains only 15–20 airway glands in wild-type (WT) mice and an abundance of glands in CF-KO mice (De Jonge, 2007; Ianowski et al., 2007). Furthermore, they demonstrated that glands from an inbred congenic *Cftr^-/-^* mouse strain responded to CCh but not to VIP or FSK similar to human CF glands (Ianowski et al., 2007). This study also documented that glands from CF mice did not secrete when they were exposed to chili oil, a treatment that produces secretion through stimulation of sensory nerves and the release of tachykinins, such as substance P. Additionally, it was demonstrated for the first time, that on tracheal SMGs of *Cftr^-/-^* mice, the substance P-stimulated fluid secretion requires CFTR (Ianowski et al., 2007).

The neuropeptide VIP is abundantly secreted around all exocrine glands including SMGs and sweat glands. It is well known to work in synergy with ACh to regulate secretions (Lundberg et al., 1980; Heinz-Erian et al., 1985, 1986). Interestingly, VIP KO mice generated in the C57BL/6 background demonstrated, *in vivo*, the central role of VIP in CFTR regulation (Chappe and Said, 2012; Alcolado et al., 2014). Originally developed to study airway diseases, such as bronchial asthma (Hamidi et al., 2006; Szema et al., 2006; Said, 2009), VIP-KO mice were used by Alcolado et al. (2014) to demonstrate the molecular link between VIP and CFTR regulation in the airways and small intestines, and observed a CF-like phenotype. The absence of VIP resulted in CFTR intracellular retention. A subsequent loss of CFTR-dependent chloride current was measured in functional assays with an Ussing chamber analysis of the small intestine *ex vivo*. In the lung, lymphocyte aggregation, increased airway secretion, alveolar thickening, and edema were observed. Inflammation and tissue damage observed in VIP-KO mice were attributed, at least in part, to the lack of CFTR-dependent secretions that rely on VIP stimulation of CFTR function. These observations support early findings of a lack of VIPergic innervation of human CF glands.

### Nasal and Tracheal Epithelium

Murine CF nasal epithelium exhibits two important characteristics of human CF airways: Na^+^ hyper-absorption and a defect in cAMP-mediated Cl^-^ secretion (Grubb and Boucher, 1999; Tarran et al., 2001). Hyper-absorption of Na^+^ was demonstrated *in vivo* with an increased baseline PD across the epithelium that was largely decreased in the presence of the ENaC blocker, amiloride (Grubb et al., 1994; Grubb and Boucher, 1999). In addition, the decreased level or absence of cAMP-stimulated Cl^-^ conductance was noticed in all murine models except for F508del *Cftr*^tm1Eur^. As mentioned above, the ∼10% residual normal CFTR expression in this model, may allow for a residual Cl^-^ permeability. In addition, nasal epithelium from *Cftr*^tm1Unc^ and *Cftr*^tm1Hsc^ demonstrates a Cl^-^ secretory response, which may be due to calcium dependent chloride channel (CACC) (Grubb and Boucher, 1999). The CACC is the prevalent Cl^-^ channel in murine tracheal epithelium (Rock et al., 2009). The gene that encodes this “pseudo” Cl^-^ channel is *TMEM16A*, and it was shown to be necessary for maintaining regular ASL levels. Studies on tracheas from *Tmem16a^-/-^* mice demonstrated more than 60% lower CACC activity and reduced MCC with mucus accumulation (Ousingsawat et al., 2009; Rock et al., 2009).

Despite bioelectric similarities, the murine nasal mucosa is composed of ∼40% olfactory epithelia and 60% RE, while the human nasal cavity is lined by more than 95% RE. However, the dominant cell type is the cilia cell, as in humans (Harkema et al., 1991; Tarran et al., 2001). In the *in vivo* analysis of *Cftr*^tm1Unc^, the nasal septal mucosa demonstrated goblet cell hyperplasia and enlargement correlated with reduced ASL height rather than changes in salt composition (Tarran et al., 2001). A study using *Cftr*^Hm1G551D^ and *Cftr^tm1Unc^–TgN^(FABRCFTR)^* mouse models was conducted to examine whether the structural changes in nasal mucosa were related to infection and inflammation. Interestingly, they detected features that are comparable with human CF tissues, such as thickening of the RE with an increased number of mucus cells. The conclusion of the study suggested that the changes observed were not a consequence of initial inflammation (Hilliard et al., 2008). Another study from Grubb et al. (2007) using *Cftr*^tm1Unc^ mice reported that an ion transport defect in CF results in progressive morphological and structural changes in the olfactory epithelium of the nasal cavity (Grubb et al., 2007).

The nasal cavity has been used extensively to investigate Cl^-^ permeability in response to corrector drug treatment by measuring the PD across the nasal epithelium. The murine nasal mucosa appears to offer an excellent model for investigating the abnormal electrophysiological profile of CF epithelial cells and testing potential corrector drugs. The murine trachea has also been used for studying cartilaginous rings alterations in *Cftr^-/-^* and F508del-CFTR mice. Structural modifications were observed in both newborn and adult CF mice independently of the CFTR absence or protein dysfunction and those changes could be indigenous (Bonvin et al., 2008).

Although the murine nasal cavity closely resembles that of humans and is easily accessible, most studies have rather used murine tracheal SMGs to examine MCC abnormalities and airway submucosal inflammation (Zahm et al., 1997; Grubb et al., 2004; Ianowski et al., 2007). Additionally, data from other animal models reported significant differences in size, density, secretory capacity and fluid secretion defects between nasal and tracheal glands. Ianowski et al. (2007) suggested that the murine trachea is an appropriate tissue for studying airway glands abnormalities in CF (Ianowski et al., 2007). Airway SMGs are found in the proximal trachea of mice. In the cartilaginous rings, CFTR expression is found at the highest levels in SMG serous tubules as reported by Engelhardt et al. (1992). Furthermore, it is well known that serous cells secrete antimicrobial proteins, such as lysozyme and lactoferrin, indicating the importance of tracheal glands in CF disease (Engelhardt et al., 1992). Nevertheless, in murine trachea, the CACC exhibits “CFTR-like” activity and protects CF mice from having the severe lung phenotype that is observed in humans. Consequently, one must ask whether murine tracheal glands are appropriate for studying secretion abnormalities in CF. Until now, there has been no consensus about it.

### Cystic Fibrosis Mice Develop Severe Postnatal Intestinal Disease

One of the earliest disease manifestations in CF is the MI in 15–20% of CF newborns and distal intestinal obstruction syndrome in 25% of adults (Zielenski et al., 1999). Moreover, CF mice are characterized by severe intestinal obstructions, including mucus accumulation, goblet cell hyperplasia, and crypt dilatation and elongation, symptoms similar to the human disease manifestations (Grubb and Boucher, 1999; Norkina et al., 2004), which develop postnatally and appear to be the hallmark for all the CF mouse models (Grubb and Gabriel, 1997; Keiser and Engelhardt, 2011) with the exception of only two mouse models (*Cftr*^tm1HGU^ and *Cftr*^tm1Eur^). The severity of the disease and the survival rate differ among genotypes (Grubb and Gabriel, 1997; Durie et al., 2004; Canale-Zambrano et al., 2007; De Lisle and Borowitz, 2013). The neonatal death rate is ∼60% for CF null mice and only 35% for F508del-CFTR. However, this percentage of MI is still high compare to only 10% in the human disease. The *Cftr*^tm1Eur^ and *Cftr*^tm2Hgu^ models, carrying the F508del and G480C mutations, respectively, exhibit intestinal alterations that are not as severe as those in the *CFTR*-KO mice (De Lisle and Borowitz, 2013). The *Cftr*^tm1G551D^ has an approximate 70% survival rate with less severe pathology than the null models (Scholte et al., 2004). A murine F508del model was generated with a replacement-gene targeting method by Colledge et al. (1995) and was suggested to be an accurate model for testing therapeutic drugs. It demonstrated severe meconium obstruction, reduced cAMP-stimulated Cl^-^ secretion, and the temperature-dependent trafficking defect that is described associated with this mutation in the human disease (Colledge et al., 1995). Tissue sections of the colon and duodenum revealed huge crypt dilation with increased mucus accumulation. As previously mentioned, the genetic background variation and low residual level of expression of normal CFTR proteins have been proposed to explain dissimilarities between models.

Studies have shown that the expression of modifier genes may also be responsible for this intestinal variability. A genetic modifier of MI was detected in chromosome 19 in humans and on mouse chromosome 7 and was shown to be involved in the disease progression in KO mice (Rozmahe et al., 1996; Zielenski et al., 1999; Gyömörey et al., 2000). Norkina et al. (2004), using a DNA microarray, observed the presence of some gene expression associated with inflammation and presence of innate immune cells, such as mast cells and neutrophils, similar to those observed in the intestine of patients with CF (Norkina et al., 2004). Amelioration of the intestinal obstruction and an increase in the survival rate was achieved by the expression of human CFTR cDNA in the intestinal track of *Cftr*^tm1Unc^ mice, under the control of the rat intestinal fatty-acid-binding protein (iFABP) gene promoter. This *Cftr^tm1Unc^–TgN*^(FABPCFTR)^ model enabled long-term studies in the intestine (De Lisle and Borowitz, 2013).

In a recent study, McHugh et al. (2018b) investigated the efficacy of linaclotide in treating intestinal pathophysiology in CF mice carrying the F508del mutation or *Cftr-*KO mice. Linaclotide is a drug that has been approved by FDA to treat chronic idiopathic constipation. This study demonstrated that this drug elevates the amount of fluid in the intestinal lumen and improves intestinal transit, thus prompting the authors to recommend linaclotide as a therapeutic option to treat CF intestinal manifestations (Than et al., 2016; McHugh et al., 2018a).

Electrophysiological studies have confirmed the ion transport abnormalities in CF mice with a similar phenotype to that of the human CF intestine: low or absence of cAMP-mediated Cl^-^ transport (Grubb and Gabriel, 1997). Moreover, they display a significant reduction in the basal PD and short-circuit current (I_sc_) (Grubb, 1999).

In summary, the CF murine intestine exhibits many pathophysiological similarities with that in humans with CF. However, it is important to categorize the different mouse models and background strains, the various techniques by which they were generated, the pathogen exposure, and the husbandry conditions during the research before evaluating the intestinal phenotype.

### Cystic Fibrosis Mice Develop Mild Pancreatic Disease

In contrast to patients with CF in whom exocrine PI is a major condition, CF mice exhibit only moderate pancreatic changes (Snouwaert et al., 1992). Durie et al. (2004) demonstrated that a long-living CF-KO mouse bred into a congenic C57BL/6 background develops pancreatic pathology similar to humans with presence of inflammatory cells and macrophages and increased acinar volume at an older age (Durie et al., 2004). Surprisingly, mice with the F508del or G551D mutation, do not show any apparent pancreatic disease (Dorin et al., 1992; Colledge et al., 1995; van Doorninck et al., 1995; Delaney et al., 1996). The milder pancreatic disease in mice is attributed first to the lower level of normal CFTR protein expression in the murine pancreas, while high CFTR expression level has been shown in humans (Gray et al., 1995), and second to differences in the CACC channel expression. The CACC is detected in both WT and CF mice alleviating the pancreatic disease progression and perhaps explaining the mild murine pancreatic pathology (Marino et al., 1991; Clarke et al., 1994; Gray et al., 1995; Keiser and Engelhardt, 2011).

### Mice Sweat Glands – An Underestimated Tissue in Cystic Fibrosis Research

Increased NaCl reabsorption abnormalities from sweat have been broadly studied with the sweat concentration being the most reliable pathophysiological marker. In mice, sweat glands are present in the palmoplantar skin (paw) (Lu and Fuchs, 2014). This tissue has largely been underutilized in CF research. In humans, sweat glands are controlled by adrenergic, cholinergic, and peptidergic innervation (Heinz-Erian et al., 1985). Heinz-Erian et al. (1985) demonstrated a reduction of VIP, in the sweat glands of patients with CF (Heinz-Erian et al., 1985). They proposed that, since VIP activates the opening of the CFTR channel, enabling the movement of water and Cl^-^ across epithelium, the lack of VIP innervation could cause low levels of water, Cl^-^ impermeability, and abnormalities involving other ions that manifest in CF disease. Sato et al. (1994) investigated changes in cholinergic and β-adrenergic responsiveness during the postnatal life and reported that the mouse sweat glands are mainly under cholinergic control and express CFTR. Sweat secretion can be monitored in response to cAMP elevating agonists with activation of either K^+^ channels, Cl^-^ channels, or both. The secretion rates are higher in older animals (over 6-weeks old) but are independent from glandular enlargement. Additionally, Chappe et al. (personal communication) examined VIP expression in sweat glands of F508del mice and found support for the murine sweat glands as a valuable tissue for studying CF disease progression. Sweat glands remain free of infection, do not exhibit morphological alterations and are easy to collect. Accordingly, their use should be further evaluated for studying CF disease development and progression.

## Porcine Models in Cystic Fibrosis Research

### Cystic Fibrosis Pigs Develop Lung Pathology Gradually

The expression of the CACC channel in parallel with the CFTR channel in some mouse organs and the inability of mice to develop “spontaneous” lung disease led investigators to examine the pathology of the CF lung using other animal models. Rogers et al. (2008b) recommended porcine airways as a possible model for CF because of their similar lung anatomy and morphological similarities with humans and identical bioelectric ion properties. Welsh et al. (2001, 2009) (Rogers et al., 2008b; Fisher et al., 2011) developed the first CF pigs with homologous recombination, utilizing adeno-associated virus (rAAV) vectors to target the *CFTR* gene in fibroblasts from fetal pigs. A somatic cell nuclear transfer of these cells into oocytes was made to generate a heterozygous male pig and through heterozygous × heterozygous breeding, *Cftr^-/-^* piglets were born (Rogers et al., 2008b).

At birth, the lungs of the *Cftr^-/-^* piglets, like those of human CF infants, are normal with no signs of pathology (Rogers et al., 2008c). However, in contrast to control animals, BAL fluid from neonatal CF piglets revealed a large number of different bacterial species (Rogers et al., 2008c; Welsh et al., 2009; Stoltz et al., 2010) and a rapid lung infection, which shows a more severe lung pathology than in patients with CF (Stoltz et al., 2010; Wine, 2010). When lungs from newborn WT and CF piglets were specifically challenged with *S. aureus*, the most common lung pathogen in infants and children with CF, only the CF piglets had more *S. aureus* than normal (Stoltz et al., 2010). That could be due to abnormalities in the pH of the ASL caused by reduced CFTR-dependent bicarbonate transport as reported by Pezzulo et al. (2012). Newborn *Cftr^-/-^* pigs were also used to examine macrophage dysfunction and to determine if their defective polarization and function are due to the absence of CFTR, or a consequence of the chronic inflammation and infection. Data from this study suggested that defective macrophages function is present at the onset of the disease and is the cause of inflammation (Paemka et al., 2017). Since the newborn CF pigs do not present signs of lung inflammation at birth, they are considered a good model for studying macrophages dysfunction prior to the beginning of the chronic inflammation.

Importantly, neonatal CF piglets have not been examined after a challenge with *P. aeruginosa*, and hence we do not know if CF pig airways are able to eradicate these bacteria. These data suggest that lungs from newborn CF pigs lack inflammation at birth but are unable to sterilize their lungs, suggesting that infection initiates inflammation (Wine, 2010).

The CF piglets revealed tracheal abnormalities, including narrowed proximal airways with thicker and more discontinuous cartilage, enlarged SMGs, and hyperplasia. Alterations in tracheal smooth muscle of F508del homozygote piglets were milder because of the residual CFTR function (Ostedgaard et al., 2011). These congenital tracheal alterations are similar to airway structural changes that occur in CF infants (Meyerholz et al., 2010a; Stoltz et al., 2015). It is suggested that the loss of CFTR produces tracheal abnormalities that may contribute to CF disease development during postnatal life (Rogers et al., 2008c; Welsh et al., 2009). However, the specific mechanisms for these abnormalities and how the absence of CFTR during development induces these defects are still unknown (Meyerholz et al., 2010a).

While mucociliary transport plays a significant role in maintaining the airways sterile, it is still unknown if in CF it is defective from birth and contributes to the beginning of the disease or if it becomes defective later, contributing to disease progression. A study from Hoegger et al. (2014a), established a new *in vivo* mucociliary transport assay (X-ray-computed tomographic-based method) with newborn pigs, which provides important insight into the pathophysiology of CF, as well as other respiratory diseases, at their early stages. The authors analyzed the movement of particles in bulk, and individually, in the large airways. They found that cilia orientation directs particles to the ventral area of the trachea and to the larynx, while the rate of individual particle’s movement was largely heterogynous. They speculate that different mucus properties may account for this heterogeneity (Hoegger et al., 2014a). Moreover, using 350 μm diameter tantalum microdisks, they found that CF SMGs secrete dense mucus strands that remained tethered and prevent the movement of those dense disks. The authors conclude that impaired mucus detachment from SMGs could be an early defect in CF (Hoegger et al., 2014b).

The development of lung disease in CF pigs is not easy to investigate because most of them die from MI that occurs in 100% of the piglets (Rogers et al., 2008c; Welsh et al., 2009; Meyerholz et al., 2010b; Stoltz et al., 2010, 2013; Olivier et al., 2015). A study from Stoltz et al. (2010), performing an ileostomy on CF pigs that survived approximately 2 months or more, demonstrated the presence of structural, bacteriological and inflammatory markers of CF. Part of the airways were characterized by the presence of macrophages, neutrophils and bacteria, similar to those in human airways. Ostedgaard et al. (2011) demonstrated that, despite residual CFTR expression, older *Cftr*^F508del/F508del^ pigs exhibited lung disease similar to humans, rendering the minimal CFTR activity observed (∼6% to WT function) insignificant for lung pathogenesis. A study from Shah et al. (2016) demonstrated that CF pigs, like humans, have ASL acidification in contrast to mice. This is thought to be due to ATP12A in absence of CFTR chloride channels. Acidification of the ASL impairs airway defense while ATP12A inhibition, which increases the pH, improves host defense properties of the ASL in CF pigs (Shah et al., 2016).

Although CF pigs recapitulate most of the human manifestations of CF, only a small number have been used for studying CF pathology, mostly due to the difficulty of keeping them alive long enough. Even after an ileostomy or cecostomy to cure MI, 60% of the piglets still had to be euthanized for gastric ulcers (Stoltz et al., 2010; Wine, 2010; Olivier et al., 2015). Long-term research still needs to be conducted to validate the data.

### Porcine Submucosal Glands – Excellent for Studying Secretion

Porcine lungs highly express CFTR proteins in the serous cells of SMGs of the cartilaginous airways, and, as in humans, exhibit the same secretion abnormalities in CF animals, with no detected morphological changes (Rogers et al., 2008c; Welsh et al., 2009). Chen et al. (2010) demonstrated a lack of cAMP-mediated Cl^-^ transport and subsequently impaired bicarbonate (HNO_3_^-^) transport with an absence of sodium hyper-absorption or reduction in ASL depth (Chen et al., 2010). This finding in CF pigs, with no signs of inflammation at birth, contradicts the hypothesis that Na^+^ hyper-absorption might trigger lung disease. The authors suggested that defects in Cl^-^ and HNO_3_^-^ initiate lung pathology and not deviations in sodium transport (Chen et al., 2010; Stoltz et al., 2015).

Ion transport abnormalities were detected in nasal airway epithelia from CF piglets with a hyperpolarized baseline transepithelial voltage (Vt), while the addition of amiloride (ENaC inhibitor) decreased the Vt in all CF piglets (Rogers et al., 2008a,c; Welsh et al., 2009). Perfusion of the apical membrane with a solution containing low Cl^-^ concentration and isoproterenol (to elevate cAMP) hyperpolarized nasal Vt in WT and heterozygous but not in *Cftr^-/-^* pigs (Rogers et al., 2008a,c; Joo et al., 2010). Lastly, perfusion with ATP to stimulate P2Y2 receptors and CACC channels produced further hyper-polarization while the CFTR inhibitor glybenclamide depolarized Vt in *Cftr*^+/+^ and *Cftr*^+/-^ but not in *Cftr^-/-^* piglets, demonstrating that a lack of CFTR channel function causes electrolyte transport abnormalities in airway epithelia (Rogers et al., 2008c; Welsh et al., 2009). The CFTR selective inhibitor CFTRinh-172 was found to be less effective with the porcine protein compared to the human CFTR (Rogers et al., 2008a). Liu et al. (2007) have reported species-specific differences in airway epithelial electrolyte transport and CFTR function (Liu et al., 2007).

### Nasal vs. Tracheal Submucosal Glands

Human CF SMGs only secrete minimally in response to agonists that increase intracellular cAMP alone (VIP and FSK) or slightly to agonists that elevate [Ca^2+^]_i_ (substance P and CCh) (Choi et al., 2007). The combination of these two types of agents produce a synergetic response that is lost in CF. Accordingly, Joo et al. (2010) showed that tracheal SMGs from CF piglets did not respond to FSK but did respond to CCh, while substance P proved to be more efficacious in CF pigs than in humans. In contrast to human SMGs, *Cftr^-/-^* pigs exhibited synergistic secretion which was attributed to CFTR-independent pathways (Joo et al., 2010). Future studies on the specific mechanisms are necessary and would clarify whether this tissue is appropriate for studying fluid secretion defects in CF disease.

Interestingly, a study from Ostedgaard et al. (2017) demonstrated that tracheal SMGs from CF pigs produce mucin 5B (MUC5B) while goblet cells produce mainly mucin 5A (MUC5AC) and moderate MUC5B. The same study suggested that these two types of mucins have different morphologic structures that account for a more effective MCC transport (Ostedgaard et al., 2017).

Cho et al. (2011) described morphological and fluid secretion differences between nasal and tracheal glands in CF piglets. In contrast to tracheal glands, nasal glands were found to be smaller and more numerous than in humans (Cho et al., 2011) with no response to FSK and a moderate response to CCh, while the synergy was almost lost (Joo et al., 2010). These findings are of high interest because, as Rogers et al. (2008c) showed, CF piglets have abnormal ion transport in nasal epithelium (Rogers et al., 2008c) which could contribute to lung pathology in CF when combined with defective fluid secretion from nasal glands.

### Intestinal Obstruction Is Fatal in Cystic Fibrosis Piglets

Meconium ileus is 100% fatal for CF pigs and a surgical ileostomy must be performed right after birth so as they can survive longer (Welsh et al., 2009; Meyerholz et al., 2010b; Stoltz et al., 2013). Accordingly, to suppress intestinal obstruction, Stoltz et al. (2013) used the rat intestinal fatty-acid-binding protein promoter (iFABP) to express WT porcine CFTR proteins (pCFTR) in *Cftr^-/-^* “gut-corrected” pigs (Stoltz et al., 2013). Their data supported the hypothesis that approximately 20% of CFTR expression was adequate to partially restore CFTR-mediated Cl^-^ transport. Similar to *Cftr^-/-^* and *Cftr*^F508*del*/F508*del*^ pigs, these transgenic animals developed pancreatic, liver, and lung disease and therefore failure to thrive (Stoltz et al., 2013). The expression of CFTR proteins in parts of the intestine, other than in ileum, might be important for improving the MI defect. Gut correction was tested only in neonatal CF pigs and hence it is not known yet if the required amount of CFTR function during adult life would be the same. Nonetheless, while surgical gut correction is not always successful, this transgenic model would be another method for making these animals live long enough (up to 12 months) to study disease progression.

Interestingly, while one would expect less disease severity in *Cftr*^F508del/F508del^ pigs compared to null animals, due to residual function of CFTR (∼6% of WT), they still show 100% penetrance of MI, as was reported by Ostedgaard et al. (2011). In general, MI in CF pigs closely resembles the disease in humans, while distinct anatomy or physiology in combination with the differences in genetic background between pigs and humans may account for the dissimilarity of penetrance rate.

### Porcine Pancreatic Pathogenesis Is Closely Similar to Humans

Cystic fibrosis pigs closely recapitulate pancreatic pathogenesis of humans CF with varying levels of disease severity among genotypes (Rogers et al., 2008c; Meyerholz et al., 2010b). After surgical correction of MI, PI develops spontaneously in these animals (Keiser and Engelhardt, 2011). The exocrine pancreas in newborn *Cftr^-/-^* pigs, as in human CF neonates, is smaller than normal, with degenerative lobules containing loose adipose tissue, mild, scattered inflammation, and dilated ducts with eosinophils, neutrophils, and macrophages. The endocrine tissue is intact, spared from the surrounding exocrine destruction with fewer acinar cells and zymogen granules (Rogers et al., 2008c; Welsh et al., 2009; Meyerholz et al., 2010b). Insulin-immunoreactive islets are mostly localized on the intact lobular tissue without alterations in the amount of insulin produced or glucagon cells. The volume and pH of pancreatic fluid are reduced in CF neonatal pigs, as in humans, with increased protein concentration (Gibson-Corley et al., 2016).

Interesting data from Abu-El-Haija et al. (2012) in fetal and newborn *Cftr^-/-^* and *Cftr*^F508del/F508del^ pigs demonstrated that tissue damage starts *in utero* and pro-inflammatory, complement cascade, pro-apoptotic, and pro-fibrotic pathways are involved (Abu-El-Haija et al., 2012). They noticed elevated activation of the apoptotic caspase-3 pathway in the CF pig pancreas whereas the α-smooth muscle actin, a myofibroblast marker and activator of early fibrosis pathways, was observed in both fetal and newborn pigs. Tissue remodeling with mucus accumulation and duct cell proliferation was observed in the neonatal and not in fetal CF pigs, indicating that these changes do not initiate disease pathogenesis but follow pancreatic damage over time. Compared to *Cftr^-/-^*, *Cftr*^F508del/F508del^ pigs exhibit slightly less severe exocrine pancreatic pathogenesis that may be attributed to the residual CFTR function (Ostedgaard et al., 2011). Future studies that investigate how much CFTR chloride channel function is necessary for the correction of pancreatic disease would be a fundamental knowledge for the progression of CF pathogenesis. Studies in adult CF pigs would be useful and would enhance our understanding of pancreatic damage in humans.

## Ferret Models in Cystic Fibrosis Research

### Comparable Lung Pathogenesis With Cystic Fibrosis Patients

The lungs of ferrets share many similarities with human lungs in terms of function and architecture, and like CF pigs, CF ferrets develop spontaneous lung infections soon after birth (Sun et al., 2010, 2014a; Keiser et al., 2015). Similar to pigs, CF ferrets were produced using homologous recombination. The first genetically designed CF ferret was described by Sun et al. (2008), utilizing rAAV-mediated targeting of the *CFTR* gene in fetal fibroblasts followed by nuclear transfer cloning (Sun et al., 2008). The *Cftr^-/-^* ferrets develop severe lung pathology within the first weeks of life, and like piglets, they are unable to eliminate bacteria. Data from the BAL fluid of newborn CF ferrets revealed various bacterial species including *Streptococcus*, *Staphylococcus* and *Enterococcus* genera while three species of *Pseudomonas* (*P. fluorescents, P. putida* and *P. fulva*) were found in low amounts (Sun et al., 2010, 2014a). In addition, macrophages from CF ferrets produce high levels of cytokines after being challenged with pathogens such as *P. aeruginosa*, *Bcc* or challenged with LPS (Bruscia and Bonfield, 2016). Interestingly, a study from Keiser et al. (2015) demonstrated differences in BAL fluid composition from CF and WT animals (Keiser et al., 2015). Levels of lactoferrin and lysozyme were especially increased in the naturally born CF ferrets in contrast to those delivered by C-section, while α_1_-antitrypsin and apolipoprotein A1 were reduced following natural birth but remained unaltered in animals delivered by C-section (Keiser et al., 2015). Moreover, proteins related to neutrophil activity were raised in naturally born CF ferrets but absent in those delivered by C-section. The authors measured levels of inflammatory cytokines (IL-1β, IL-8, and TNF-a). They did not find any alteration between C-sectioned CF and non-CF ferrets while the newborn CF animals exhibited lower levels of IL-1β, but IL-8 and TNF-α were higher, similar to infants with CF.

Thus, they concluded that inflammatory dissimilarities in CF ferrets may start *in utero* and result in excessive inflammation during birth, following bacterial exposure (Keiser et al., 2015). These data suggest that innate immunity and inflammatory pathways change under sterile and non-sterile conditions, making these animals a valuable model for studying the early onset and progression of CF disease.

Adult CF ferrets do not show abnormal inflammatory response. However, they have other similarities in lung pathology with humans, such as airway obstruction, thick mucus accumulation, and inflammation (Sun et al., 2014a).

### Postnatal Development of Submucosal Glands

In ferrets, as in humans, the CFTR protein is highly expressed in serous cells of SMGs lining their cartilaginous rings. Newborn ferrets do not have ciliated cells or SMGs, but they develop them abundantly a few months after birth (Sun et al., 2014a; Keiser et al., 2015). A study from Keiser et al. (2015) on newborn *Cftr^-/-^* ferrets reported that tracheal MCC was similar to non-CF ferrets, while ASL height was decreased. Only 1–3 weeks after birth, when MCC rate slightly increases, CF related MCC abnormalities would have an impact on the lung pathology (Keiser et al., 2015). Additionally, Sun et al. (2014a) demonstrated a seven-fold decrease in MCC in adult compared to juvenile *Cftr^-/-^* ferrets that was attributed to an age-dependent ENaC hyperactivity (Sun et al., 2014a). The unique characteristic of ferret lungs to develop SMGs postnatally suggests the presence of CFTR-independent mechanisms that cause the early lung inflammatory profile and future investigations will be valuable.

### Ferret Trachea Is Appropriate for Studying Gland Secretion

Adult ferrets have an abundance of tracheal glands, and like humans, they depend on Cl^-^ and HCO_3_^-^ for secretion (CFTR-dependent). In adult WT ferret tracheas, Cho et al. (2010) revealed a reduced secretion rate by blocking Cl^-^ or HCO_3_^-^ in response to VIP or FSK. Additionally, CCh-induced MCC was highly decreased when blocking the Na^+^-K^+^-2Cl^-^ (NKCC) co-transporter with bumetanide. The authors showed synergistic secretion when low concentrations of agonists that elevate [cAMP]_i_ were combined with low concentrations of agonists that elevate [Ca^2+^]_i._ Neonatal ferret tracheas contain few cells that express high levels of CFTR since SMGs are not yet developed. Studies on newborn *Cftr^-/-^* ferrets have shown abnormal fluid secretion from their tracheas in response to FSK and CCh, with higher reduction to FSK stimulation, similar to data from human proximal CF airways (Sun et al., 2010).

As CF ferrets have a similar airway morphology to humans, they are a good model for studying CF pathogenesis. They develop human-like lung disease and CFTR-dependent secretion in trachea, albeit the amiloride-sensitive transepithelial PD was unaltered between CF and non-CF ferret tracheas. However, research in CF ferret fluid secretion is spare, and while it seems a promising animal model, our knowledge has been limited until now.

### Intestinal Disease Is More Severe in Ferrets Compared to Humans

Newborn CF ferrets, like CF piglets, present a higher incidence of MI than seen in human infants with CF. Between 50% and 100% of CF ferrets develop MI and die within 36 h due to intestinal obstruction and sepsis (Fisher et al., 2011). Most of CF ferrets that survive MI still fail to thrive soon after birth because of malabsorption. Compared to other animals, they do not have caecum and therefore have less time to digest (Sun et al., 2010). A study from Sun et al. (2014b) in CF ferrets over a month old reported that the small intestine was characterized by a reduction in the height of the villi and mucus accumulation in the crypt with signs of inflammation within the lamina propria and into the epithelium (Sun et al., 2014b). A metagenomic deep sequencing analysis revealed the exacerbation of *Streptococcus*, *Enterococcus*, and *Escherichia* in CF ferret feces, similar to what occurs in CF infants (Sun et al., 2014b). A gut-corrected transgenic model that expresses the *CFTR* gene under the control of the rat fatty acid-binding protein promoter (iFABP), is shown to correct MI in newborn *Cftr^-/-^* ferrets (Sun et al., 2010). The generation of a F508del-CFTR ferret would be useful to increase our knowledge of CF with the most common mutation.

### Pancreatic Insufficiency Occurs the First Months of Life

Newborn *Cftr^-/-^* ferrets exhibit mild pancreatic pathology, similar to CF human infants, and are characterized by swollen acini and ducts with eosinophilic zymogen material, moderate alterations and pancreatic lesions observed in approximately 75% of *Cftr^-/-^* ferrets (Keiser and Engelhardt, 2011; Sun et al., 2014b). Olivier et al. (2012) demonstrated that neonatal CF ferrets exhibited elevated apoptosis in exocrine ducts and acini suggesting that pancreatic inflammation starts very early in life. Within the first month of life and before adulthood, the pancreas undergoes rapid tissue deterioration with destruction of acini, duct dilatation and evidence of inflammatory cells (neutrophils, macrophages and lymphocytes) (Olivier et al., 2012). Similar to patients with CF, 85% of adult CF ferrets present a loss of exocrine pancreas with fibrosis and islet remodeling. Interestingly, a small percentage of CF ferrets, like patients with CF, are born PS with light pancreatic pathology while they have normal growth and fecal elastase-1 (EL1) levels (Sun et al., 2014b). This finding demonstrates that, as in patients with CF, modifier genes could be responsible for this small percentage of PS. Additionally, adult CF ferrets exhibit disorganized islets, characterized by large clusters that contain an abundance of β and α cells as occurs in the pancreas of humans with CF (Olivier et al., 2012). In general, CF ferrets share many features of human pancreatic disease, and their ability to exhibit variability of disease severity at birth and in adulthood makes them an excellent model for testing potential therapies and examining the specific mechanisms for PI in CF.

## Lung and Intestine Observations in a Recent Cystic Fibrosis Rat Model

The first CF rat model was generated by Tuggle et al. (2014) with the use of the zinc-finger endonuclease technique. Rats are an appealing model for studying airway defects in CF because they share many characteristics consistent with humans. In contrast to mice, where SMGs are only located at the proximal trachea, rats SMGs extend to bronchi as in humans. The *Cftr^-/-^* rats, shortly after birth, exhibit congenital tracheal defects with a reduction in the cartilage and tracheal gland area (Tuggle et al., 2014). Nasal PD measurements demonstrated loss of Cl^-^ permeability in response to FSK, suggesting that the absence of CFTR chloride channels changes electrophysiological features in the rat (Tuggle et al., 2014). Additionally, young CF rats revealed reduced amounts of ASL in distal airways, similar to humans, while BAL fluid was free of inflammatory markers. The decreased ASL levels were not accompanied with Na^+^ hyper-absorption suggesting that ASL regulation was CFTR and not ENaC dependent (Tuggle et al., 2014). Young CF rats did not show rapid pulmonary pathology progression, while the sterile environmental conditions under which they were kept may have had an effect (Tuggle et al., 2014). Future studies that will examine disease progression in adult CF rats housed in a natural environment will clarify the role of ASL depletion in airway obstruction. The presence of an increased number of dilated mucus cells was noticed in the proximal nasal epithelium, adding another similarity with the human pathology.

A study from Tipirneni et al. (2017) investigated nasal septal epithelial cultures from *Cftr^-/-^* rats and demonstrated that they would be a useful model for *in vitro* sinosal ion transport studies. The authors demonstrated that those cell cultures have comparable *Cftr* gene expression and nasal electrophysiological properties to nasal septal epithelial cultures from human and mice, while being more responsive to ion transport modulators (Tipirneni et al., 2017). Moreover, a recent study from Birket et al. (2018) demonstrated that the development of the SMGs is delayed in the *Cftr^-/-^* rats but only when the SMGs are fully developed, elevated mucus viscosity and impaired mucociliary transport were observed. Hyperacidic pH and changes in the periciliary layer, however, happen before SMGs develop. Importantly, blocking bicarbonate transport increased mucus viscosity. Additional studies on secretion abnormalities in tracheal glands using CF rats will add important information.

In addition, 70% of *Cftr^-/-^* rats mortality is due to intestinal obstruction between weaning and 42 days after birth in contrast to piglets and ferrets that suffered from severe intestinal obstruction at birth. Their small intestine was characterized by crypt dilation and mucus accumulation (Tuggle et al., 2014).

While CF rats do not spontaneously develop lung or pancreatic disease during their first 6-weeks of life, they are a good model for investigating bone growth and its direct link to CFTR, without the presence of other disease manifestations such as airway disease. Accordingly, Stalvey et al. (2017) examined the role of CFTR loss on bone growth using young *Cftr^-/-^* rats by measuring their femur length, bone histomorphometry and cartilaginous growth plates as well as the concentration of serum IGF-I. Deficiency in IGF-I can cause changes in cartilage growth. They found that in absence of the *CFTR* gene, rats show reduced growth and bone content. Also, IGF-I concentration in the serum of *Cftr^-/-^* rats (a marker also used in non-CF children with bone disease) is decreased, which may explain changes in the growth plate (Stalvey et al., 2017).

In general, the *Cftr^-/-^* rat is a promising model for long-term studies of CF disease manifestations. Although it is in the early stages, this model could be the base for generating CF rats with disease-causing mutations that would allow investigations of CFTR protein trafficking or gating defects and CFTR correctors and potentiators.

## Cystic Fibrosis Zebrafish – a Unique Model of Pancreatic Disease Progression in All Developmental Stages

Zebrafish have been valued as an outstanding tiny research model for studying human diseases. They are inexpensive, and can be generated in large numbers, while requiring minimal housing and handling conditions (Phillips and Westerfield, 2014). Moreover, zebrafish CFTR chloride channels responds similarly to most agonists and antagonists of the human CFTR due to its high homology. Data have demonstrated that the absence of CFTR in zebrafish hinders the development of Kupffer’s vesicle (KV), a basic ciliated organ, which determines lateralization in an early developmental zebrafish stage, emphasizing the importance of CFTR function in fluid secretion (Navis and Bagnat, 2015).

The first zebrafish CF model was produced by Navis and Bagnat (2015) using transgenic zebrafish mutant lines. They reported that CFTR was localized on the apical membrane of pancreatic ducts and investigated the absence of CFTR in zebrafish pancreas during various developmental stages suggesting comparable disease pathology as in humans. They reported that most of CF larval zebrafish started to die approximately 10 days post fertilization (dpf), suggesting that pancreatic dysfunction starts early in life. By 16 dpf, pancreatic tissue was partially damaged and neutrophil presence was slightly evident while by 20 dpf, the pancreas was remarkably disrupted. Pancreatic ducts contained mucus, and they were encircled by fibrotic tissue instead of acinar and were highly infiltrated with neutrophils. Additionally, the islets appeared widespread, abundant and diminished in size. The CF zebrafish model closely resembles pancreatic pathogenesis in human adults with CF suggesting that a mild PI may start early in life. Additional studies examining the specific mechanisms that cause pancreatic disease early in life, the role of the inflammatory factors in disease progression, and the ion transport abnormalities using the zebrafish model would be very valuable tools for pancreatic pathogenesis in CF.

## Sheep CF Model – a Brief Description of an Attractive Undervalued Model

The sheep model is not a commonly used model for studying CF disease compared to mice, pigs and ferrets, respectively. However, at the molecular level, the sheep *CFTR* gene is 91% homologous with the human *CFTR* while at protein level, they have 95% resemblance (Harris, 1997). Many researchers have used an ovine model for studying respiratory diseases such as asthma and chronic obstructive pulmonary disease (COPD) since anatomically, functionally and electrophysiologically sheep airways parallels those of humans (Abraham, 2008; Allen et al., 2009). It has been utilized for examining secretion abnormalities, testing the effect of small molecules as a potential therapy for lung disease and for gene therapy (Cai et al., 2015). Apart from the lung pathology, CFTR protein expression in sheep pancreas mirrors human levels. As a large animal, in combination with its long lifespan, it could be appropriate for therapeutic trials. Generation of a CF ovine model utilizing somatic cell cloning was not successful in the past due to failure of homologous recombination in sheep fibroblasts. In 2018, Fan et al. generated the first *Cftr^-/-^* sheep model using the CRISPR/Cas9 genome editing and somatic cell nuclear transfer methods. The newborn *Cftr^-/-^* sheep demonstrated severe intestinal obstruction similar to the MI noticed in approximately 15% of human babies with CF. The pancreas was characterized by hypoplasia and fibrosis similar to humans with CF. This pancreatic hypoplasia was common in both KO and heterozygous sheep. Interestingly, lungs of newborn *Cftr^-/-^* sheep had minimal signs of disease which is an important feature that enables investigations of early disease progression (Fan et al., 2018). Production of a CF sheep model gave rise to a new chapter of large animals as useful models for novel therapeutic approaches.

## Discussion

Despite recent research progress, our understanding of CF pathogenesis is still incomplete. Many questions remain unanswered and CF continues to be a fatal disease. Cell-culture models have been shown to be useful for pre-clinical trials but they lack the structural complexity of intact organs (Scholte et al., 2006; Rogers et al., 2008c; Cutting, 2015). Much of the knowledge on CF pathophysiology that we have today derives from animal models and especially from the wide variability of gene-targeted mouse models. Most of the studies repeatedly note that mice models, in contrast to pigs and ferrets, do not spontaneously develop lung disease as do humans. However, depending on the strain, such as congenic models with the C57BL/6 background, they have the advantage of surviving longer from intestinal disease and are susceptible to lung infection with pathogens, mainly the most persistent *P. aeruginosa*. Pigs and ferrets exhibit similar lung function and architecture with humans and at birth they lack inflammation, and they are unable to eradicate bacteria (Rogers et al., 2008c; Stoltz et al., 2010; Sun et al., 2010; Wine, 2010; Ostedgaard et al., 2011). The disadvantage of these models is the difficulty of keeping them alive long enough since, approximately 48 h after birth they die from intestinal obstruction and a surgical ileostomy is mandatory but not always successful. Consequently, while most of the studies report a lung pathology similar to that in patients with CF, they are only based on experiments that have been conducted in very few animals, mostly newborn (Rogers et al., 2008c; Joo et al., 2010; Stoltz et al., 2010; Sun et al., 2010, 2014a; Wine, 2010; Ostedgaard et al., 2011; Keiser et al., 2015).

Mice SMGs, contrary to those in rats that extend into the bronchi, are confined to the trachea, but they are abundant and identical in structure to those in humans. Despite the bioelectric similarities of murine nasal SMGs with those in humans, tracheal glands are widely utilized due to the highest expression levels of CFTR proteins, whereas studies on *Cftr^-/-^* mice have demonstrated secretion defects. Tracheal glands from CF pigs also exhibit secretion defects but they do not completely lose synergy as mice or newborn CF ferrets do (Joo et al., 2010). Secretion defects are restricted only in newborn CF ferrets and CF pigs, in contrast to mice, while studies in CF ferret nasal glands have not been done yet (Sun et al., 2010, 2014a; Keiser et al., 2015). The dominance of the CACC channel in mice airways, intestine and pancreas, in parallel with CFTR proteins, alleviate the severity of the disease pathogenesis compared to other models. Intestinal pathology can be fatal for some CF mice, causing death a few days after birth or around the time of weaning (Davidson and Dorin, 2001). The disease severity varies among strains and although it is more severe than in humans, CF mice intestine still exhibit the same pathophysiological abnormalities, and they are considered a good model for studying human intestinal disease.

In addition, MI is fatal for both CF piglets and ferrets with a penetrance of 100% and 50–100%, respectively, while the occurrence in human infants with CF is only moderate at 15–20%. The generation of transgenic mice, pigs, and ferrets with tissue-corrected *CFTR* gene expression under the control of the iFABP gene promoter alleviates intestinal obstruction with no effects on the other organs. Interestingly, milder pancreatic disease is observed in CF mice, while CF pigs and ferrets that survived from MI demonstrated human-like disease. However, studies in adult pigs and ferrets are limited. The first CF zebrafish model enabled the examination of pancreatic pathogenesis in all developmental stages, demonstrating that a mild pancreatic disease starts early in life (Navis and Bagnat, 2015).

Although human CF is characterized by salty sweat, eccrine sweat glands are underestimated in the above CF animal models. Pigs use mud baths to cool their body (Rogers et al., 2008a) as they have very few sweat glands, which are in the nose, while ferrets do not sweat at all. Importantly, mice have sweat glands in their paws, this is a promising tissue for studying CF disease which should be further evaluated.

## Conclusion

In conclusion, while various animal models present different advantages for studying different aspects of the disease, there is no single animal model available yet that completely replicates the complexity of CF in humans. Each model presents pros and cons and combining findings from various organs studied in different models provides important clues for our understanding of the CF disease pathogenesis and progression.

## Author Contributions

AS wrote the paper. All authors contributed to the review of appropriate literature and preparation of the manuscript.

## Conflict of Interest Statement

The authors declare that the research was conducted in the absence of any commercial or financial relationships that could be construed as a potential conflict of interest.

## Abbreviations

rAAV: adeno-associated virus
AA: amino acid
ATP: adenosine triphosphate
ASL: airway surface liquid
BAL: bacterial alveolar lavage
*Bcc*: *Burkholderia cepacia* complex
CACC: Ca^2+^-activated Cl^-^ channel
CFTR: cystic fibrosis transmembrane conductance regulator protein
*CFTR*: cystic fibrosis transmembrane conductance regulator gene
CF: cystic fibrosis
ENaC: epithelial sodium channel
iFABP: intestinal fatty-acid binding protein promoter
[Ca^2+^]_I_: intracellular Ca^2+^
KO: knockout
LPS: lipopolysaccharides
MI: meconium ileus
MUC5AC: mucin 5AC
MUC5B: mucin 5B
MCC: mucociliary clearance
PI: pancreatic insufficiency, PS, pancreatic sufficient
PD: potential difference
RE: respiratory epithelia
I_sc_: short-circuit current
SMGs: submucosal glands, Vt, transepithelial voltage
VIP: vasoactive intestinal peptide
WT: wild type

## References

[B1] Abdulrahman B. A. Khweek A. A. Akhter A. Caution K. Kotrange S. Abdelaziz D. H. (2011). Autophagy stimulation by rapamycin suppresses lung inflammation and infection by Burkholderia cenocepacia in a model of cystic fibrosis. *Autophagy* 7 1359–1370. 10.4161/auto.7.11.17660 21997369 PMC3359483

[B2] Abraham W. M. (2008). Modeling of asthma, COPD and cystic fibrosis in sheep. *Pulm. Pharmacol. Ther.* 21 743–754. 10.1016/j.pupt.2008.01.010 18339565

[B3] Abu-El-Haija M. Ramachandran S. Meyerholz D. K. Abu-El-Haija M. Griffin M. Giriyappa R. L. (2012). Pancreatic damage in fetal and newborn cystic fibrosis pigs involves the activation of inflammatory and remodeling pathways. *Am. J. Pathol.* 181 499–507. 10.1016/j.ajpath.2012.04.024 22683312 PMC3409440

[B4] Alcolado N. G. Conrad D. J. Poroca D. Li M. Alshafie W. Chappe F. G. (2014). Cystic fibrosis transmembrane conductance regulator dysfunction in VIP knockout mice. *Am. J. Physiol. Cell Physiol.* 307 C195–C207. 10.1152/ajpcell.00293.2013 24898584 PMC4101624

[B5] Allen J. E. Bischof R. J. Chang H. Y. S. Hirota J. A. Hirst S. J. Inman M. D. (2009). Animal models of airway inflammation and airway smooth muscle remodelling in asthma. *Pulm. Pharmacol. Ther.* 22 455–465. 10.1016/j.pupt.2009.04.001 19393759

[B6] Andersen D. H. (1938). Cystic fibrosis of the pancreas and its relation to celiac disease: a clinical and pathologic study. *Am. J. Dis. Child.* 56 344–399. 10.1001/archpedi.1938.01980140114013

[B7] Andersson C. Zaman M. M. Jones A. B. Freedman S. D. (2008). Alterations in immune response and PPAR/LXR regulation in cystic fibrosis macrophages. *J. Cyst. Fibros.* 7 68–78. 10.1016/j.jcf.2007.05.004 17889625

[B8] Birket S. E. Davis J. M. Fernandez C. M. Tuggle K. L. Oden A. M. Chu K. K. (2018). Development of an airway mucus defect in the cystic fibrosis rat. *JCI Insight* 10.1172/jci.insight.97199 [Epub ahead of print]. 29321377 PMC5821204

[B9] Bonvin E. Le Rouzic P. Bernaudin J. Cottart C. Vandebrouck C. Crié A. (2008). Congenital tracheal malformation in cystic fibrosis transmembrane conductance regulator deficient mice. *J. Physiol.* 586 3231–3243. 10.1113/jphysiol.2008.150763 18450781 PMC2538793

[B10] Borthwick D. W. West J. D. Keighren M. A. Flockhart J. H. Innes B. A. Dorin J. R. (1999). Murine submucosal glands are clonally derived and show a cystic fibrosis gene–dependent distribution pattern. *Am. J. Respir. Cell Mol. Biol.* 20 1181–1189. 10.1165/ajrcmb.20.6.3475 10340937

[B11] Bruscia E. M. Bonfield T. L. (2016). Cystic fibrosis lung immunity: the role of the macrophage. *J. Innate Immun.* 8 550–563. 10.1159/000446825 27336915 PMC5089923

[B12] Bruscia E. M. Zhang P. X. Ferreira E. Caputo C. Emerson J. W. Tuck D. (2009). Macrophages directly contribute to the exaggerated inflammatory response in cystic fibrosis transmembrane conductance regulator-/- mice. *Am. J. Respir. Cell Mol. Biol.* 40 295–304. 10.1165/rcmb.2008-0170OC 18776130 PMC2645527

[B13] Bruscia E. M. Zhang P. X. Satoh A. Caputo C. Medzhitov R. Shenoy A. (2011). Abnormal trafficking and degradation of TLR4 underlie the elevated inflammatory response in cystic fibrosis. *J. Immunol.* 186 6990–6998. 10.4049/jimmunol.1100396 21593379 PMC3111054

[B14] Cai Z. Palmai-Pallag T. Khuituan P. Mutolo M. J. Boinot C. Liu B. (2015). Impact of the F508del mutation on ovine CFTR, a Cl- channel with enhanced conductance and ATP-dependent gating. *J. Physiol.* 593 2427–2446. 10.1113/JP270227 25763566 PMC4461407

[B15] Canale-Zambrano J. C. Poffenberger M. C. Cory S. M. Humes D. G. Haston C. K. (2007). Intestinal phenotype of variable-weight cystic fibrosis knockout mice. *Am. J. Physiol. Gastrointest. Liver Physiol.* 293 G222–G229. 10.1152/ajpgi.00405.2006 17615178

[B16] Chappe V. Said S. I. (2012). “VIP as a corrector of CFTR trafficking and membrane stability,” in *Cystic Fibrosis-Renewed Hopes through Research*, ed. Sriramulu D. (London: InTech).

[B17] Chen J. Stoltz D. A. Karp P. H. Ernst S. E. Pezzulo A. A. Moninger T. O. (2010). Loss of anion transport without increased sodium absorption characterizes newborn porcine cystic fibrosis airway epithelia. *Cell* 143 911–923. 10.1016/j.cell.2010.11.029 21145458 PMC3057187

[B18] Cho H. Joo N. S. Wine J. J. (2011). Defective fluid secretion from submucosal glands of nasal turbinates from CFTR-/-and CFTRΔF508/ΔF508 Pigs. *PLoS One* 6:e24424. 10.1371/journal.pone.0024424 21935358 PMC3164206

[B19] Cho H. J. Joo N. S. Wine J. J. (2010). Mucus secretion from individual submucosal glands of the ferret trachea. *Am. J. Physiol. Lung Cell. Mol. Physiol.* 299 L124–L136. 10.1152/ajplung.00049.2010 20435689 PMC2904090

[B20] Choi J. Y. Joo N. S. Krouse M. E. Wu J. V. Robbins R. C. Ianowski J. P. (2007). Synergistic airway gland mucus secretion in response to vasoactive intestinal peptide and carbachol is lost in cystic fibrosis. *J. Clin. Invest.* 117 3118–3127. 10.1172/JCI31992 17853942 PMC1974867

[B21] Clarke L. L. Grubb B. R. Yankaskas J. R. Cotton C. U. McKenzie A. Boucher R. C. (1994). Relationship of a non-cystic fibrosis transmembrane conductance regulator-mediated chloride conductance to organ-level disease in Cftr(-/-) mice. *Proc. Natl. Acad. Sci. U.S.A.* 91 479–483. 10.1073/pnas.91.2.479 7507247 PMC42972

[B22] Colledge W. H. Abella B. S. Southern K. W. Ratcliff R. Jiang C. Cheng S. H. (1995). Generation and characterization of a ΔF508 cystic fibrosis mouse model. *Nat. Genet.* 10 445–452. 10.1038/ng0895-445 7545494

[B23] Cowley E. A. Wang C. G. Gosselin D. Radzioch D. Eidelman D. H. (1997). Mucociliary clearance in cystic fibrosis knockout mice infected with *Pseudomonas aeruginosa*. *Eur. Respir. J.* 10 2312–2318. 10.1183/09031936.97.10102312 9387959

[B24] Cutting G. R. (2015). Cystic fibrosis genetics: from molecular understanding to clinical application. *Nat. Rev. Genet.* 16 45–56. 10.1038/nrg3849 25404111 PMC4364438

[B25] Davidson D. J. Dorin J. R. (2001). The CF mouse: an important tool for studying cystic fibrosis. *Expert Rev. Mol. Med.* 3 1–27. 14987374 10.1017/S1462399401002551

[B26] Davis P. B. (2006). Cystic fibrosis since 1938. *Am. J. Respir. Crit. Care Med.* 173 475–482. 10.1164/rccm.200505-840OE 16126935

[B27] De Jonge H. R. (2007). Cystic fibrosis mice rehabilitated for studies of airway gland dysfunction. *J. Physiol.* 580 7–8. 10.1113/jphysiol.2007.129361 17317740 PMC2075432

[B28] De Lisle R. C. Borowitz D. (2013). The cystic fibrosis intestine. *Cold Spring Harb. Perspect. Med.* 3:a009753. 10.1101/cshperspect.a009753 23788646 PMC3753720

[B29] Delaney S. J. Alton E. Smith S. N. Lunn D. P. Farley R. Lovelock P. (1996). Cystic fibrosis mice carrying the missense mutation G551D replicate human genotype / phenotype correlations. *EMBO J.* 15 955–963. 10.1002/j.1460-2075.1996.tb00432.x 8605891 PMC449990

[B30] Dorin J. Stevenson B. Fleming S. Alton E. Dickinson P. Porteous D. (1994). Long-term survival of the exon 10 insertional cystic fibrosis mutant mouse is a consequence of low level residual wild-type Cftr gene expression. *Mamm. Genome* 5 465–472. 10.1007/BF00369314 7949729

[B31] Dorin J. R. Dickinson P. Alton E. W. Smith S. N. Geddes D. M. Stevenson B. J. (1992). Cystic fibrosis in the mouse by targeted insertional mutagenesis. *Nature* 359 211–215. 10.1038/359211a0 1382232

[B32] Durie P. R. Forstner G. G. (1989). Pathophysiology of the exocrine pancreas in cystic fibrosis. *J. R. Soc. Med.* 82(Suppl. 16), 2–10. PMC1291913 2657051

[B33] Durie P. R. Kent G. Phillips M. J. Ackerley C. A. (2004). Characteristic multiorgan pathology of cystic fibrosis in a long-living cystic fibrosis transmembrane regulator knockout murine model. *Am. J. Pathol.* 164 1481–1493. 10.1016/S0002-9440(10)63234-8 15039235 PMC1615340

[B34] Ellsworth R. E. Jamison D. C. Touchman J. W. Chissoe S. L. Braden Maduro V. V. Bouffard G. G. (2000). Comparative genomic sequence analysis of the human and mouse cystic fibrosis transmembrane conductance regulator genes. *Proc. Natl. Acad. Sci. U.S.A.* 97 1172–1177. 10.1073/pnas.97.3.1172 10655503 PMC15558

[B35] Engelhardt J. F. Yankaskas J. R. Ernst S. A. Yang Y. Marino C. R. Boucher R. C. (1992). Submucosal glands are the predominant site of CFTR expression in the human bronchus. *Nat. Genet.* 2 240–248. 10.1038/ng1192-240 1285365

[B36] Fan Z. Perisse I. V. Cotton C. U. Regouski M. Meng Q. Domb C. (2018). A sheep model of cystic fibrosis generated by CRISPR/Cas9 disruption of the CFTR gene. *JCI Insight* 10.1172/jci.insight.123529 [Epub ahead of print]. 30282831 PMC6237476

[B37] Festini F. Buzzetti R. Bassi C. Braggion C. Salvatore D. Taccetti G. (2006). Isolation measures for prevention of infection with respiratory pathogens in cystic fibrosis: a systematic review. *J. Hosp. Infect.* 64 1–6. 10.1016/j.jhin.2006.02.021 16835001

[B38] Fisher J. T. Liu X. Yan Z. Luo M. Zhang Y. Zhou W. (2012). Comparative processing and function of human and ferret cystic fibrosis transmembrane conductance regulator. *J. Biol. Chem.* 287 21673–21685. 10.1074/jbc.M111.336537 22570484 PMC3381131

[B39] Fisher J. T. Zhang Y. Engelhardt J. F. (2011). Comparative biology of cystic fibrosis animal models. *Methods Mol. Biol.* 742 311–334. 10.1007/978-1-61779-120-8_19 21547741 PMC3617920

[B40] Gibson L. E. Cooke R. E. (1959). A test for concentration of electrolytes in sweat in cystic fibrosis of the pancreas utilizing pilocarpine by iontophoresis. *Pediatrics* 23 545–549. 13633369

[B41] Gibson-Corley K. N. Meyerholz D. K. Engelhardt J. F. (2016). Pancreatic pathophysiology in cystic fibrosis. *J. Pathol.* 238 311–320. 10.1002/path.4634 26365583 PMC4699289

[B42] Gosselin D. Stevenson M. M. Cowley E. A. Griesenbach U. Eidelman D. H. BoulÉ M. (1998). Impaired ability of CFTR knockout mice to control lung infection with *Pseudomonas aeruginosa*. *Am. J. Respir. Crit. Care Med.* 157 1253–1262. 10.1164/ajrccm.157.4.9702081 9563748

[B43] Gray M. A. Winpenny J. P. Verdon B. McAlroy H. Argent B. E. (1995). Chloride channels and cystic fibrosis of the pancreas. *Biosci. Rep.* 15 531–541. 10.1007/BF01204355 9156582

[B44] Grubb B. R. (1999). Ion transport across the normal and CF neonatal murine intestine. *Am. J. Physiol.* 277(1 Pt 1), G167–G174. 10.1152/ajpgi.1999.277.1.G167 10409164

[B45] Grubb B. R. Boucher R. C. (1999). Pathophysiology of gene-targeted mouse models for cystic fibrosis. *Physiol. Rev.* 79 S193–S214. 10.1152/physrev.1999.79.1.S193 9922382

[B46] Grubb B. R. Gabriel S. E. (1997). Intestinal physiology and pathology in gene-targeted mouse models of cystic fibrosis. *Am. J. Physiol.* 273(2 Pt 1), G258–G266. 10.1152/ajpgi.1997.273.2.G258 9277402

[B47] Grubb B. R. Jones J. H. Boucher R. C. (2004). Mucociliary transport determined by in vivo microdialysis in the airways of normal and CF mice. *Am. J. Physiol. Lung Cell. Mol. Physiol.* 286 L588–L595. 10.1152/ajplung.00302.2003 14633516

[B48] Grubb B. R. Rogers T. D. Kulaga H. M. Burns K. A. Wonsetler R. L. Reed R. R. (2007). Olfactory epithelia exhibit progressive functional and morphological defects in CF mice. *Am. J. Physiol. Cell Physiol.* 293 C574–C583. 10.1152/ajpcell.00106.2007 17428842

[B49] Grubb B. R. Vick R. N. Boucher R. C. (1994). Hyperabsorption of Na^+^ and raised Ca(2+)-mediated Cl- secretion in nasal epithelia of CF mice. *Am. J. Physiol.* 266(5 Pt 1), C1478–C1483. 10.1152/ajpcell.1994.266.5.C1478 7515571

[B50] Guilbault C. Martin P. Houle D. Boghdady M. Guiot M. Marion D. (2005). Cystic fibrosis lung disease following infection with *Pseudomonas aeruginosa* in Cftr knockout mice using novel non-invasive direct pulmonary infection technique. *Lab. Anim.* 39 336–352. 10.1258/0023677054306944 16004694

[B51] Guilbault C. Novak J. P. Martin P. Boghdady M. L. Saeed Z. Guiot M. C. (2006). Distinct pattern of lung gene expression in the Cftr-KO mice developing spontaneous lung disease compared with their littermate controls. *Physiol. Genomics* 25 179–193. 10.1152/physiolgenomics.00206.2005 16418321

[B52] Gyömörey K. Rozmahel R. Bear C. E. (2000). Amelioration of intestinal disease severity in cystic fibrosis mice is associated with improved chloride secretory capacity. *Pediatr. Res.* 48 731–734. 10.1203/00006450-200012000-00005 11102538

[B53] Hamidi S. Szema A. Lyubsky S. Dickman K. Degene A. Mathew S. (2006). Clues to VIP function from knockout mice. *Ann. N. Y. Acad. Sci.* 1070 5–9. 10.1196/annals.1317.035 16888146

[B54] Harkema J. Mariassy A. George J. S. Hyde D. Plopper C. (1991). Epithelial cells of the conducting airways: a species comparison. *Lung Biol. Health Dis.* 55 3–39.

[B55] Harris A. (1997). Towards an ovine model of cystic fibrosis. *Hum. Mol. Genet.* 6 2191–2193. 10.1093/hmg/6.13.2191 9361022

[B56] Hasty P. O’Neal W. K. Liu K. Morris A. P. Bebok Z. Shumyatsky G. B. (1995). Severe phenotype in mice with termination mutation in exon 2 of cystic fibrosis gene. *Somat. Cell Mol. Genet.* 21 177–187. 10.1007/BF02254769 7482032

[B57] Hasty P. Ramírez-Solis R. Krumlauf R. Bradley A. (1991). Introduction of a subtle mutation into the Hox-2.6 locus in embryonic stem cells. *Nature* 350 243–246. 10.1038/350243a0 1672446

[B58] Heeckeren A. Walenga R. Konstan M. W. Bonfield T. Davis P. B. Ferkol T. (1997). Excessive inflammatory response of cystic fibrosis mice to bronchopulmonary infection with *Pseudomonas aeruginosa*. *J. Clin. Invest.* 100 2810–2815. 10.1172/JCI119828 9389746 PMC508486

[B59] Heinz-Erian P. Dey R. D. Flux M. Said S. I. (1985). Deficient vasoactive intestinal peptide innervation in the sweat glands of cystic fibrosis patients. *Science* 229 1407–1408. 10.1126/science.4035357 4035357

[B60] Heinz-Erian P. Paul S. Said S. I. (1986). Receptors for vasoactive intestinal peptide on isolated human sweat glands. *Peptides* 7 151–154. 10.1016/0196-9781(86)90178-6 3018693

[B61] Hilliard T. N. Zhu J. Farley R. Escudero-Garcia S. Wainwright B. J. Jeffery P. K. (2008). Nasal abnormalities in cystic fibrosis mice independent of infection and inflammation. *Am. J. Respir. Cell Mol. Biol.* 39 19–25. 10.1165/rcmb.2007-0284OC 18239192

[B62] Hoegger M. J. Awadalla M. Namati E. Itani O. A. Fischer A. J. Tucker A. J. (2014a). Assessing mucociliary transport of single particles in vivo shows variable speed and preference for the ventral trachea in newborn pigs. *Proc. Natl. Acad. Sci. U.S.A.* 111 2355–2360. 10.1073/pnas.1323633111 24474805 PMC3926068

[B63] Hoegger M. J. Fischer A. J. McMenimen J. D. Ostedgaard L. S. Tucker A. J. Awadalla M. A. (2014b). Impaired mucus detachment disrupts mucociliary transport in a piglet model of cystic fibrosis. *Science* 345 818–822. 10.1126/science.1255825 25124441 PMC4346163

[B64] Ianowski J. P. Choi J. Y. Wine J. J. Hanrahan J. W. (2007). Mucus secretion by single tracheal submucosal glands from normal and cystic fibrosis transmembrane conductance regulator knockout mice. *J. Physiol.* 580 301–314. 10.1113/jphysiol.2006.123653 17204498 PMC2075436

[B65] Joo N. S. Cho H. J. Khansaheb M. Wine J. J. (2010). Hyposecretion of fluid from tracheal submucosal glands of CFTR-deficient pigs. *J. Clin. Invest.* 120 3161–3166. 10.1172/JCI43466 20739758 PMC2929738

[B66] Keiser N. W. Birket S. E. Evans I. A. Tyler S. R. Crooke A. K. Sun X. (2015). Defective innate immunity and hyperinflammation in newborn cystic fibrosis transmembrane conductance regulator–knockout ferret lungs. *Am. J. Respir. Cell Mol. Biol.* 52 683–694. 10.1165/rcmb.2014-0250OC 25317669 PMC4491130

[B67] Keiser N. W. Engelhardt J. F. (2011). New animal models of cystic fibrosis: what are they teaching us? *Curr. Opin. Pulm. Med.* 17 478–483. 10.1097/MCP.0b013e32834b14c9 21857224 PMC3596000

[B68] Kent G. Iles R. Bear C. E. Huan L. J. Griesenbach U. McKerlie C. (1997). Lung disease in mice with cystic fibrosis. *J. Clin. Invest.* 100 3060–3069. 10.1172/JCI119861 9399953 PMC508519

[B69] Lavelle G. M. White M. M. Browne N. McElvaney N. G. Reeves E. P. (2016). Animal models of cystic fibrosis pathology: phenotypic parallels and divergences. *Biomed Res. Int.* 2016:5258727. 10.1155/2016/5258727 27340661 PMC4908263

[B70] Li C. Wu Y. Riehle A. Ma J. Kamler M. Gulbins E. (2017). *Staphylococcus aureus* survives in cystic fibrosis macrophages, forming a reservoir for chronic pneumonia. *Infect. Immun.* 85:e00883–816. 10.1128/IAI.00883-16 28289144 PMC5400852

[B71] Liu X. Luo M. Zhang L. Ding W. Yan Z. Engelhardt J. F. (2007). Bioelectric properties of chloride channels in human, pig, ferret, and mouse airway epithelia. *Am. J. Respir. Cell Mol. Biol.* 36 313–323. 10.1165/rcmb.2006-0286OC 17008635 PMC1894945

[B72] Liu F. Zhang Z. Csanády L. Gadsby D. C. Chen J. (2017). Molecular structure of the human CFTR ion channel. *Cell* 169 85–95. 10.1016/j.cell.2017.02.024 28340353

[B73] Lu C. Fuchs E. (2014). Sweat gland progenitors in development, homeostasis, and wound repair. *Cold Spring Harb. Perspect. Med.* 4:a015222. 10.1101/cshperspect.a015222 24492848 PMC3904096

[B74] Luciani A. Villella V. R. Esposito S. Brunetti-Pierri N. Medina D. Settembre C. (2010). Defective CFTR induces aggresome formation and lung inflammation in cystic fibrosis through ROS-mediated autophagy inhibition. *Nat. Cell Biol.* 12 863–875. 10.1038/ncb2090 20711182

[B75] Lundberg J. M. Anggard A. Fahrenkrug J. Hokfelt T. Mutt V. (1980). Vasoactive intestinal polypeptide in cholinergic neurons of exocrine glands: functional significance of coexisting transmitters for vasodilation and secretion. *Proc. Natl. Acad. Sci. U.S.A.* 77 1651–1655. 10.1073/pnas.77.3.1651 6103537 PMC348555

[B76] Marino C. R. Matovcik L. M. Gorelick F. S. Cohn J. A. (1991). Localization of the cystic fibrosis transmembrane conductance regulator in pancreas. *J. Clin. Invest.* 88 712–716. 10.1172/JCI115358 1713921 PMC295422

[B77] McHugh D. R. Cotton C. U. Moss F. J. Vitko M. Valerio D. M. Kelley T. J. (2018a). Linaclotide improves gastrointestinal transit in cystic fibrosis mice by inhibiting sodium/hydrogen exchanger 3. *Am. J. Physiol. Gastrointest. Liver Physiol.* 10.1152/ajpgi.00261.2017 [Epub ahead of print]. 30118317 PMC9925117

[B78] McHugh D. R. Steele M. S. Valerio D. M. Miron A. Mann R. J. LePage D. F. (2018b). A G542X cystic fibrosis mouse model for examining nonsense mutation directed therapies. *PLoS One* 13:e0199573. 10.1371/journal.pone.0199573 29924856 PMC6010256

[B79] McMorran B. J. Palmer J. S. Lunn D. P. Oceandy D. Costelloe E. O. Thomas G. R. (2001). G551D CF mice display an abnormal host response and have impaired clearance of *Pseudomonas* lung disease. *Am. J. Physiol. Lung Cell. Mol. Physiol.* 281 L740–L747. 10.1152/ajplung.2001.281.3.L740 11504703

[B80] Meyer M. Huaux F. Gavilanes X. Van Den Brûle S. Lebecque P. Lo Re S. (2009). Azithromycin reduces exaggerated cytokine production by M1 alveolar macrophages in cystic fibrosis. *Am. J. Respir. Cell Mol. Biol.* 41 590–602. 10.1165/rcmb.2008-0155OC 19244203

[B81] Meyerholz D. K. Stoltz D. A. Namati E. Ramachandran S. Pezzulo A. A. Smith A. R. (2010a). Loss of cystic fibrosis transmembrane conductance regulator function produces abnormalities in tracheal development in neonatal pigs and young children. *Am. J. Respir. Crit. Care Med.* 182 1251–1261. 10.1164/rccm.201004-0643OC 20622026 PMC3001264

[B82] Meyerholz D. K. Stoltz D. A. Pezzulo A. A. Welsh M. J. (2010b). Pathology of gastrointestinal organs in a porcine model of cystic fibrosis. *Am. J. Pathol.* 176 1377–1389. 10.2353/ajpath.2010.090849 20110417 PMC2832157

[B83] Navis A. Bagnat M. (2015). Loss of Cftr function leads to pancreatic destruction in larval zebrafish. *Dev. Biol.* 399 237–248. 10.1016/j.ydbio.2014.12.034 25592226 PMC4765326

[B84] Norkina O. Kaur S. Ziemer D. De Lisle R. C. (2004). Inflammation of the cystic fibrosis mouse small intestine. *Am. J. Physiol. Gastrointest. Liver Physiol.* 286 G1032–G1041. 10.1152/ajpgi.00473.2003 14739145

[B85] Olivier A. K. Gibson-Corley K. N. Meyerholz D. K. (2015). Animal models of gastrointestinal and liver diseases. animal models of cystic fibrosis: gastrointestinal, pancreatic, and hepatobiliary disease and pathophysiology. *Am. J. Physiol. Gastrointest. Liver Physiol.* 308 G459–G471. 10.1152/ajpgi.00146.2014 25591863 PMC4360044

[B86] Olivier A. K. Yi Y. Sun X. Sui H. Liang B. Hu S. (2012). Abnormal endocrine pancreas function at birth in cystic fibrosis ferrets. *J. Clin. Invest.* 122 3755–3768. 10.1172/JCI60610 22996690 PMC3534166

[B87] O’Neal W. K. Hasty P. McCray P. B. Jr. Casey B. RiveraPérez J. Welsh M. J. (1993). A severe phenotype in mice with a duplication of exon 3 in the cystic fibrosis locus. *Hum. Mol. Genet.* 2 1561–1569. 10.1093/hmg/2.10.1561 7505691

[B88] Ostedgaard L. S. Meyerholz D. K. Chen J. H. Pezzulo A. A. Karp P. H. Rokhlina T. (2011). The DeltaF508 mutation causes CFTR misprocessing and cystic fibrosis-like disease in pigs. *Sci. Transl. Med.* 3:74ra24. 10.1126/scitranslmed.3001868 21411740 PMC3119077

[B89] Ostedgaard L. S. Moninger T. O. McMenimen J. D. Sawin N. M. Parker C. P. Thornell I. M. (2017). Gel-forming mucins form distinct morphologic structures in airways. *Proc. Natl. Acad. Sci. U.S.A.* 114 6842–6847. 10.1073/pnas.1703228114 28607090 PMC5495256

[B90] Ostedgaard L. S. Rogers C. S. Dong Q. Randak C. O. Vermeer D. W. Rokhlina T. (2007). Processing and function of CFTR-deltaF508 are species-dependent. *Proc. Natl. Acad. Sci. U.S.A.* 104 15370–15375. 10.1073/pnas.0706974104 17873061 PMC1976592

[B91] Ousingsawat J. Martins J. R. Schreiber R. Rock J. R. Harfe B. D. Kunzelmann K. (2009). Loss of TMEM16A causes a defect in epithelial Ca^2+^-dependent chloride transport. *J. Biol. Chem.* 284 28698–28703. 10.1074/jbc.M109.012120 19679661 PMC2781414

[B92] Paemka L. McCullagh B. N. Alaiwa M. H. A. Stoltz D. A. Dong Q. Randak C. O. (2017). Monocyte derived macrophages from CF pigs exhibit increased inflammatory responses at birth. *J. Cyst. Fibros.* 16 471–474. 10.1016/j.jcf.2017.03.007 28377087 PMC5515361

[B93] Pezzulo A. A. Tang X. X. Hoegger M. J. Alaiwa M. H. A. Ramachandran S. Moninger T. O. (2012). Reduced airway surface pH impairs bacterial killing in the porcine cystic fibrosis lung. *Nature* 487 109–113. 10.1038/nature11130 22763554 PMC3390761

[B94] Phillips J. B. Westerfield M. (2014). Zebrafish models in translational research: tipping the scales toward advancements in human health. *Dis. Models Mech.* 7 739–743. 10.1242/dmm.015545 24973743 PMC4073263

[B95] Ratcliff R. Evans M. J. Cuthbert A. W. MacVinish L. J. Foster D. Anderson J. R. (1993). Production of a severe cystic fibrosis mutation in mice by gene targeting. *Nat. Genet.* 4 35–41. 10.1038/ng0593-35 7685652

[B96] Riordan J. R. Rommens J. M. Kerem B. Alon N. Rozmahel R. Grzelczak Z. (1989). Identification of the cystic fibrosis gene: cloning and characterization of complementary DNA. *Science* 245 1066–1073. 10.1126/science.2475911 2475911

[B97] Rock J. R. O’Neal W. K. Gabriel S. E. Randell S. H. Harfe B. D. Boucher R. C. (2009). Transmembrane protein 16A (TMEM16A) is a Ca^2+^-regulated Cl- secretory channel in mouse airways. *J. Biol. Chem.* 284 14875–14880. 10.1074/jbc.C109.000869 19363029 PMC2685669

[B98] Rogers C. S. Abraham W. M. Brogden K. A. Engelhardt J. F. Fisher J. T. McCray P. B. Jr. (2008a). The porcine lung as a potential model for cystic fibrosis. *Am. J. Physiol. Lung Cell. Mol. Physiol.* 295 L240–L263. 10.1152/ajplung.90203.2008 18487356 PMC2519845

[B99] Rogers C. S. Hao Y. Rokhlina T. Samuel M. Stoltz D. A. Li Y. (2008b). Production of CFTR-Null and CFTR-DeltaF508 heterozygous pigs by adeno-associated virus-mediated gene targeting and somatic cell nuclear transfer. *J. Clin. Invest.* 118 1571–1577. 10.1172/JCI34773 18324337 PMC2265103

[B100] Rogers C. S. Stoltz D. A. Meyerholz D. K. Ostedgaard L. S. Rokhlina T. Taft P. J. (2008c). Disruption of the CFTR gene produces a model of cystic fibrosis in newborn pigs. *Science* 321 1837–1841. 10.1126/science.1163600 18818360 PMC2570747

[B101] Rommens J. M. Iannuzzi M. C. Kerem B. Drumm M. L. Melmer G. Dean M. (1989). Identification of the cystic fibrosis gene: chromosome walking and jumping. *Science* 245 1059–1065. 10.1126/science.2772657 2772657

[B102] Rozmahe R. Wilschanski M. Matin A. Plyte S. Oliver M. Auerbach W. (1996). Modulation of disease severity in cystic fibrosis transmembrane conductance regulator deficient mice by a secondary genetic factor. *Nat. Genet.* 12 280–287. 10.1038/ng0396-280 8589719

[B103] Said S. I. (2009). Animal models of airway hyperresponsiveness. *Eur. Respir. J.* 33 217–218. 10.1183/09031936.00131008 19118233

[B104] Salinas D. Haggie P. M. Thiagarajah J. R. Song Y. Rosbe K. Finkbeiner W. E. (2005). Submucosal gland dysfunction as a primary defect in cystic fibrosis. *FASEB J.* 19 431–433. 10.1096/fj.04-2879fje 15596485

[B105] Sato K. Cavallin S. Sato K. T. Sato F. (1994). Secretion of ions and pharmacological responsiveness in the mouse paw sweat gland. *Clin. Sci.* 86 133–139. 8143423 10.1042/cs0860133

[B106] Scholte B. J. Colledge W. H. Wilke M. de Jonge H. (2006). Cellular and animal models of cystic fibrosis, tools for drug discovery. *Drug Discov. Today Dis. Models* 3 251–259. 10.1016/j.ddmod.2006.09.003

[B107] Scholte B. J. Davidson D. J. Wilke M. De Jonge H. R. (2004). Animal models of cystic fibrosis. *J. Cyst. Fibros.* 3 183–190. 10.1016/j.jcf.2004.05.039 15463956

[B108] Shah V. S. Meyerholz D. K. Tang X. X. Reznikov L. Alaiwa M. A. Ernst S. E. (2016). Airway acidification initiates host defense abnormalities in cystic fibrosis mice. *Science* 351 503–507. 10.1126/science.aad5589 26823428 PMC4852973

[B109] Snouwaert J. N. Brigman K. K. Latour A. M. Malouf N. N. Boucher R. C. Smithies O. (1992). An animal model for cystic fibrosis made by gene targeting. *Science* 257 1083–1088. 10.1126/science.257.5073.1083 1380723

[B110] Sorio C. Montresor A. Bolomini-Vittori M. Caldrer S. Rossi B. Dusi S. (2016). Mutations of cystic fibrosis transmembrane conductance regulator gene cause a monocyte-selective adhesion deficiency. *Am. J. Respir. Crit. Care Med.* 193 1123–1133. 10.1164/rccm.201510-1922OC 26694899

[B111] Stalvey M. S. Havasi V. Tuggle K. L. Wang D. Birket S. Rowe S. M. (2017). Reduced bone length, growth plate thickness, bone content, and IGF-I as a model for poor growth in the CFTR-deficient rat. *PLoS One* 12:e0188497. 10.1371/journal.pone.0188497 29190650 PMC5708703

[B112] Stoltz D. A. Meyerholz D. K. Pezzulo A. A. Ramachandran S. Rogan M. P. Davis G. J. (2010). Cystic fibrosis pigs develop lung disease and exhibit defective bacterial eradication at birth. *Sci. Transl. Med.* 2:29ra31. 10.1126/scitranslmed.3000928 20427821 PMC2889616

[B113] Stoltz D. A. Meyerholz D. K. Welsh M. J. (2015). Origins of cystic fibrosis lung disease. *N. Engl. J. Med.* 372 351–362. 10.1056/NEJMra1300109 25607428 PMC4916857

[B114] Stoltz D. A. Rokhlina T. Ernst S. E. Pezzulo A. A. Ostedgaard L. S. Karp P. H. (2013). Intestinal CFTR expression alleviates meconium ileus in cystic fibrosis pigs. *J. Clin. Invest.* 123 2685–2693. 10.1172/JCI68867 23676501 PMC3668832

[B115] Sun X. Olivier A. K. Liang B. Yi Y. Sui H. Evans T. I. (2014a). Lung phenotype of juvenile and adult cystic fibrosis transmembrane conductance regulator–knockout ferrets. *Am. J. Respir. Cell Mol. Biol.* 50 502–512. 10.1165/rcmb.2013-0261OC 24074402 PMC4068938

[B116] Sun X. Olivier A. K. Yi Y. Pope C. E. Hayden H. S. Liang B. (2014b). Gastrointestinal pathology in juvenile and adult CFTR-knockout ferrets. *Am. J. Pathol.* 184 1309–1322. 10.1016/j.ajpath.2014.01.035 24637292 PMC4005986

[B117] Sun X. Sui H. Fisher J. T. Yan Z. Liu X. Cho H. J. (2010). Disease phenotype of a ferret CFTR-knockout model of cystic fibrosis. *J. Clin. Invest.* 120 3149–3160. 10.1172/JCI43052 20739752 PMC2929732

[B118] Sun X. Yan Z. Yi Y. Li Z. Lei D. Rogers C. S. (2008). Adeno-associated virus-targeted disruption of the CFTR gene in cloned ferrets. *J. Clin. Invest.* 118 1578–1583. 10.1172/JCI34599 18324338 PMC2263147

[B119] Szema A. M. Hamidi S. A. Lyubsky S. Dickman K. G. Mathew S. Abdel-Razek T. (2006). Mice lacking the VIP gene show airway hyperresponsiveness and airway inflammation, partially reversible by VIP. *Am. J. Physiol. Lung Cell. Mol. Physiol.* 291 L880–L886. 10.1152/ajplung.00499.2005 16782752

[B120] Tarran R. Grubb B. Parsons D. Picher M. Hirsh A. Davis C. (2001). The CF salt controversy: in vivo observations and therapeutic approaches. *Mol. Cell* 8 149–158. 10.1016/S1097-2765(01)00286-6 11511368

[B121] Tata F. Stanier P. Wicking C. Halford S. Kruyer H. Lench N. J. (1991). Cloning the mouse homolog of the human cystic fibrosis transmembrane conductance regulator gene. *Genomics* 10 301–307. 10.1016/0888-7543(91)90312-3 1712752

[B122] Than B. L. N. Linnekamp J. F. Starr T. K. Largaespada D. A. Rod A. Zhang Y. (2016). CFTR is a tumor suppressor gene in murine and human intestinal cancer. *Oncogene* 35 4179–4187. 10.1038/onc.2015.483 26751771 PMC4940277

[B123] Tipirneni K. E. Cho D. Y. Skinner D. F. Zhang S. Mackey C. Lim D. J. (2017). Characterization of primary rat nasal epithelial cultures in CFTR knockout rats as a model for CF sinus disease. *Laryngoscope* 127 E384–E391. 10.1002/lary.26720 28771736 PMC5654659

[B124] Trezise A. E. Szpirer C. Buchwald M. (1992). Localization of the gene encoding the cystic fibrosis transmembrane conductance regulator (CFTR) in the rat to chromosome 4 and implications for the evolution of mammalian chromosomes. *Genomics* 14 869–874. 10.1016/S0888-7543(05)80107-7 1282491

[B125] Tuggle K. L. Birket S. E. Cui X. Hong J. Warren J. Reid L. (2014). Characterization of defects in ion transport and tissue development in cystic fibrosis transmembrane conductance regulator (CFTR)-knockout rats. *PLoS One* 9:e91253. 10.1371/journal.pone.0091253 24608905 PMC3946746

[B126] van der Doef H. P. Kokke F. T. van der Ent C. K. Houwen R. H. (2011). Intestinal obstruction syndromes in cystic fibrosis: meconium ileus, distal intestinal obstruction syndrome, and constipation. *Curr. Gastroenterol. Rep.* 13 265–270. 10.1007/s11894-011-0185-9 21384135 PMC3085752

[B127] van Doorninck J. H. French P. J. Verbeek E. Peters R. Morreau H. Bijman J. (1995). A mouse model for the cystic fibrosis delta F508 mutation. *EMBO J.* 14 4403–4411. 10.1002/j.1460-2075.1995.tb00119.x 7556083 PMC394531

[B128] Van Heeckeren A. M. Schluchter M. D. Xue W. Davis P. B. (2006). Response to acute lung infection with mucoid *Pseudomonas aeruginosa* in cystic fibrosis mice. *Am. J. Respir. Crit. Care Med.* 173 288–296. 10.1164/rccm.200506-917OC 16272448 PMC2662931

[B129] Welsh M. J. Ramsey B. W. Accurso F. Cutting G. R. Scriver C. R. Beaudet A. L. (2001). *The Metabolic and Molecular Basis of Inherited Disease*, 8th Edn New York, NY: McGraw-Hill, 5121–5189.

[B130] Welsh M. J. Rogers C. S. Stoltz D. A. Meyerholz D. K. Prather R. S. (2009). Development of a porcine model of cystic fibrosis. *Trans. Am. Clin. Climatol. Assoc.* 120 149–162. 19768173 PMC2744522

[B131] Welsh M. J. Smith A. E. (1993). Molecular mechanism of CFTR chloride channel dysfunction in cystic fibrosis. *Cell* 73 1251–1254. 10.1016/0092-8674(93)90353-R 7686820

[B132] Wilke M. Buijs-Offerman R. M. Aarbiou J. Colledge W. H. Sheppard D. N. Touqui L. (2011). Mouse models of cystic fibrosis: phenotypic analysis and research applications. *J. Cyst. Fibros.* 10 S152–S171. 10.1016/S1569-1993(11)60020-9 21658634

[B133] Wilschanski M. Novak I. (2013). The cystic fibrosis of exocrine pancreas. *Cold Spring Harb. Perspect. Med.* 3:a009746. 10.1101/cshperspect.a009746 23637307 PMC3633181

[B134] Wine J. J. (2010). The development of lung disease in cystic fibrosis pigs. *Sci. Transl. Med.* 2:29s20. 10.1126/scitranslmed.3001130 20427819

[B135] Wong P. Y. (1998). CFTR gene and male fertility. *Mol. Hum. Reprod.* 4 107–110. 10.1093/molehr/4.2.107 9542966

[B136] Zahm J. M. Gaillard D. Dupuit F. Hinnrasky J. Porteous D. Dorin J. R. (1997). Early alterations in airway mucociliary clearance and inflammation of the *Lamina propria* in CF mice. *Am. J. Physiol.* 272(3 Pt 1), C853–C859. 10.1152/ajpcell.1997.272.3.C853 9124520

[B137] Zeiher B. G. Eichwald E. Zabner J. Smith J. J. Puga A. P. McCray P. B. (1995). A mouse model for the delta F508 allele of cystic fibrosis. *J. Clin. Invest.* 96 2051–2064. 10.1172/JCI118253 7560099 PMC185844

[B138] Zhou L. Dey C. R. Wert S. E. DuVall M. D. Frizzell R. A. Whitsett J. A. (1994). Correction of lethal intestinal defect in a mouse model of cystic fibrosis by human CFTR. *Science* 266 1705–1708. 10.1126/science.7527588 7527588

[B139] Zhou Z. Duerr J. Johannesson B. Schubert S. C. Treis D. Harm M. (2011). The ENaC-overexpressing mouse as a model of cystic fibrosis lung disease. *J. Cyst. Fibros.* 10 S172–S182. 10.1016/S1569-1993(11)60021-0 21658636

[B140] Zielenski J. Corey M. Rozmahel R. Markiewicz D. Aznarez I. Casals T. (1999). Detection of a cystic fibrosis modifier locus for meconium ileus on human chromosome 19q13. *Nat. Genet.* 22 128–129. 10.1038/9635 10369249

